# Spontaneous movement of a retrotransposon generated genic dominant male sterility providing a useful tool for rice breeding

**DOI:** 10.1093/nsr/nwad210

**Published:** 2023-08-07

**Authors:** Conghao Xu, Yifeng Xu, Zhengji Wang, Xiaoyu Zhang, Yuying Wu, Xinyan Lu, Hongwei Sun, Lei Wang, Qinglu Zhang, Qinghua Zhang, Xianghua Li, Jinghua Xiao, Xu Li, Mingfu Zhao, Yidan Ouyang, Xianbo Huang, Qifa Zhang

**Affiliations:** National Key Laboratory of Crop Genetic Improvement and National Centre of Plant Gene Research (Wuhan), Hubei Hongshan Laboratory, Huazhong Agricultural University, Wuhan 430070, China; Ningde Inspection and Testing Centre for Agricultural Product Quality and Safety, Ningde 352100, China; National Key Laboratory of Crop Genetic Improvement and National Centre of Plant Gene Research (Wuhan), Hubei Hongshan Laboratory, Huazhong Agricultural University, Wuhan 430070, China; National Key Laboratory of Crop Genetic Improvement and National Centre of Plant Gene Research (Wuhan), Hubei Hongshan Laboratory, Huazhong Agricultural University, Wuhan 430070, China; National Key Laboratory of Crop Genetic Improvement and National Centre of Plant Gene Research (Wuhan), Hubei Hongshan Laboratory, Huazhong Agricultural University, Wuhan 430070, China; National Key Laboratory of Crop Genetic Improvement and National Centre of Plant Gene Research (Wuhan), Hubei Hongshan Laboratory, Huazhong Agricultural University, Wuhan 430070, China; National Key Laboratory of Crop Genetic Improvement and National Centre of Plant Gene Research (Wuhan), Hubei Hongshan Laboratory, Huazhong Agricultural University, Wuhan 430070, China; National Key Laboratory of Crop Genetic Improvement and National Centre of Plant Gene Research (Wuhan), Hubei Hongshan Laboratory, Huazhong Agricultural University, Wuhan 430070, China; National Key Laboratory of Crop Genetic Improvement and National Centre of Plant Gene Research (Wuhan), Hubei Hongshan Laboratory, Huazhong Agricultural University, Wuhan 430070, China; National Key Laboratory of Crop Genetic Improvement and National Centre of Plant Gene Research (Wuhan), Hubei Hongshan Laboratory, Huazhong Agricultural University, Wuhan 430070, China; National Key Laboratory of Crop Genetic Improvement and National Centre of Plant Gene Research (Wuhan), Hubei Hongshan Laboratory, Huazhong Agricultural University, Wuhan 430070, China; National Key Laboratory of Crop Genetic Improvement and National Centre of Plant Gene Research (Wuhan), Hubei Hongshan Laboratory, Huazhong Agricultural University, Wuhan 430070, China; National Key Laboratory of Crop Genetic Improvement and National Centre of Plant Gene Research (Wuhan), Hubei Hongshan Laboratory, Huazhong Agricultural University, Wuhan 430070, China; Fujian Academy of Agricultural Sciences, Fuzhou 350018, China; National Key Laboratory of Crop Genetic Improvement and National Centre of Plant Gene Research (Wuhan), Hubei Hongshan Laboratory, Huazhong Agricultural University, Wuhan 430070, China; Sanming Institute of Agricultural Sciences, Shaxian 365509, China; National Key Laboratory of Crop Genetic Improvement and National Centre of Plant Gene Research (Wuhan), Hubei Hongshan Laboratory, Huazhong Agricultural University, Wuhan 430070, China

**Keywords:** *Oryza sativa*, dominant male sterility, ribosome-inactivating protein, retrotransposon

## Abstract

Male sterility in plants provides valuable breeding tools in germplasm innovation and hybrid crop production. However, genetic resources for dominant genic male sterility, which hold great promise to facilitate breeding processes, are extremely rare in natural germplasm. Here we characterized the Sanming Dominant Genic Male Sterility in rice and identified the gene *SDGMS* using a map-based cloning approach. We found that spontaneous movement of a 1978-bp long terminal repeat (LTR) retrotransposon into the promoter region of the *SDGMS* gene activates its expression in anther tapetum, which causes abnormal programmed cell death of tapetal cells resulting in dominant male sterility. *SDGMS* encodes a ribosome inactivating protein showing N-glycosidase activity. The activation of *SDGMS* triggers transcription reprogramming of genes responsive to biotic stress leading to a hypersensitive response which causes sterility. The results demonstrate that an ectopic gene activation by transposon movement can give birth to a novel trait which enriches phenotypic diversity with practical utility.

## INTRODUCTION

Male sterility is a widespread phenomenon in the plant kingdom. According to the genetic causes, male sterility can be classified as cytoplasmic male sterility and nuclear (also referred to as genic) male sterility [1]. Cytoplasmic male sterility is caused by mutations of genes in the cytoplasmic genomes, mostly mitochondria, which can be restored by nuclear restorer gene(s). Genic male sterility results from mutations of genes in the nuclear genomes, which may either be genetically recessive or dominant. Although male sterility is unfavourable to plants per se, these genetic resources provide vital breeding tools in hybrid seed production and breeding processes in many crops [2–6]. In the past decades cytoplasmic male sterility and environmentally inducible recessive genic male sterility have been widely exploited for the development of hybrid crops which have greatly boosted global food production [1,7,8]. However, dominant genic male sterility, which may also have the potential to make crucial contributions to both plant science research and crop genetic improvement, has been under explored [9].

Crossing (also referred to as hybridization) is the first and essential procedure in both breeding programs and genetic studies. Hand emasculation is the first step in hybridization of self-pollinating species, which is highly labour-intensive and economically costly especially in large breeding programs. Introduction of dominant genic male sterility can greatly reduce or even eliminate the need for hand emasculation which may revolutionize breeding processes.

However, genetic resources for dominant genic male sterility are extremely rare in natural germplasm, and, so far, only a few cases have been reported in crops [10–12]. In wheat, the insertion of a terminal-repeat retrotransposon in a miniature element in the promoter of *Ms2* activates its anther-specific expression and is therefore responsible for sterility in Taigu dominant genic male sterility [13,14]. In rapeseed, the male-sterile allele *MS5^b^* acts as a dominant suppressor of the maintainer allele *MS5^c^* to induce genic male sterility [15,16]. In maize, a single amino acid change in *Ms44* abolishes protein processing and impedes the secretion of protein from tapetal cells into the locule, resulting in dominant male sterility [17]. Due to the lack of natural genetic resources, transgenic technology has also been explored in order to create a dominant male-sterility system by premature expression of *ZmMs7* in maize by an anther-specific promoter *p5126* [18]. Transgenic dominant male sterile rice was also generated using the *barnase* gene expressed by the tapetum-specific promoter *BoA9* [19].

The Sanming Dominant Genic Male Sterile (SDGMS) Rice was first found in an F_2_ population of a cross between SE21S and Basmati370 named after the Sanming Institute of Agricultural Science [20]. Male sterility is controlled by a dominant gene that maps to a 99-kb interval on chromosome 8 [21]. This SDGMS line shows stable complete male sterility and practically is not affected by the environment, which is highly useful in breeding. In this study, we identified the *SDGMS* gene, the first dominant male sterility gene in rice, using a map-based cloning approach. We found that spontaneous movement of a retrotransposon activates *SDGMS* expression and generates dominant male sterility. Our work demonstrates a mechanism of gene activation which supplies genetic novelty and phenotypic diversity. Our study also provides a promising tool for rice breeding programs.

## RESULTS

### A naturally occurring dominant genic male sterile mutant

The SDGMS mutant was obtained from an F_2_ population of a cross between SE21S and Basmati370 (Supplementary Fig. S1a) [20]. Three near-isogenic lines (NILs), 938(*SDGMS*), ZS97(*SDGMS*) and NIP(*SDGMS*), which contained chromosomal segments with the *SDGMS* gene (in a heterozygous state) from the SDGMS mutant in the genetic background of 938 (a mutant from 93–11), Zhenshan 97 (ZS97) and Nipponbare (NIP), were developed using marker-assisted selection (Supplementary Fig. S1b). All three pairs of NILs grow normally during the vegetative stage; the NILs(*SDGMS*) showed complete male sterility with small and pale anthers producing no pollen, whereas the NILs(*sdgms*) showed normal fertility (Supplementary Fig. S2a). In addition, all three NILs could cause stable dominant male sterility when crossed with male-fertile parents, showing a 1:1 segregation ratio of male sterile and fertile plants in the next generation (Supplementary Table S1).

We characterized the cellular abnormality of *SDGMS* anther development by anther transverse sections. Compared to the wild type, *SDGMS* anthers had no observable defects before the microspore mother cell stage (MMC). During the meiosis stage, the wild-type tapetal cells and middle-layer cells became thinner and condensed and gradually degenerated before microspore formation (Supplementary Fig. S2b). In contrast, the tapetal cells and middle-layer cells of *SDGMS* anthers did not undergo degradation and eventually displayed a defective 4-layer anther wall producing no pollen (Supplementary Fig. S2b). These results showed that the abnormal abortion of anther locules was the main cause of male sterility.

A terminal deoxynucleotidyl transferase-mediated dUTP nick-end labelling (TUNEL) assay showed that in the wild-type anthers, a positive TUNEL signal was detected in tapetal and middle-layer cells during the meiosis stage. The middle layer became invisible after microspore release, and the positive signal of tapetal cells continued before the microspores were formed (Supplementary Fig. S2b). While an abnormally strong TUNEL signal was detected in *SDGMS* tapetum cells during the early meiosis stage (EM) and disappeared abruptly during the late meiosis stage (LM), no TUNEL signal was observed in middle-layer cells (Supplementary Fig. S2b), suggesting abnormal PCD of tapetal cells. Thus, the *SDGMS* anthers develop normally at MMC and defects occurred subsequently.

### Map-based cloning of *SDGMS*

**Figure 1.**
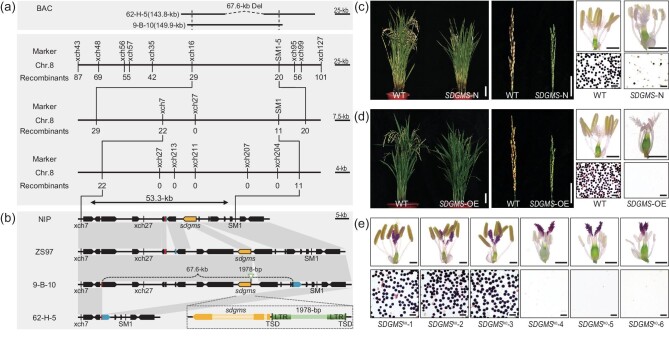
Map-based cloning and functional analysis of *SDGMS*. (a) Mapping of *SDGMS* to a 53.3-kb region on chromosome 8 (chr. 8). The distances are based on the NIP genome sequence. BAC indicates the location of two BAC clones, the dashed line indicates the 67.6-kb deletion. The horizontal lines indicate fragments of chromosome 8. The markers used for map-based cloning are indicated above the horizontal lines. (b) Comparative sequence analysis of genomic fragments between *SDGMS* and *sdgms* genotypes. Annotated genes in the mapping region are based on ZS97 genome (http://rice.hzau.edu.cn/rice_rs2/). The tip of the pentagon indicates the 3’ end of the genes. The orange colour pentagons indicate the *sdgms* and the green triangle indicates the 1978-bp insertion. In the diagram at lower right corner, the orange colour indicates the exon, and the light yellow colour indicates the intron of *sdgms*. The dark green indicates long terminal repeat (LTR) and TSD indicates the target site duplications (5-bp, CTTCT). (c) Whole plants, panicles, anthers and pollen grains stained with I_2_-KI of the wild-type and *SDGMS*-N positive plants at the maturity stage. Scale bar for plants = 10 cm, scale bar for panicles = 5 cm, scale bar for anther = 2 mm, scale bar for pollen grains = 50 μm. (d) Whole plants, panicles, anther and pollen grains stained with I_2_-KI of the wild-type and *SDGMS-*OE positive plants at the maturity stage. Scale bar for plants = 10 cm, scale bar for panicles = 5 cm, scale bar for anthers = 2 mm, scale bar for pollen grains = 50 μm. (e) Anthers and pollen grains of different versions of *SDGMS^ko^*mutants stained with I_2_-KI. The scale bar for anthers = 2 mm and the scale bar for pollen grains = 50 μm. (See online supplementary material for a colour version of this figure.)

To identify the gene responsible for the dominant male sterility, we planted a large BC_7_F_1_ population (8241 individuals) from 938(*SDGMS*) and mapped the *SDGMS* locus to a 53-kb region that contains 11 predicted genes in the NIP genome (http://rice.uga.edu/) (Fig. 1a). Because of complex variation in this genomic region, we constructed a bacterial artificial chromosome (BAC) library of genomic DNA from 938(*SDGMS*), consisting of 36 480 clones with an average DNA insert size of 110 kb. The library was screened with the markers xch43, xch7 and xch95. Two overlapping BAC clones covering the target genomic region, 62-H-5 with the *sdgms* genotype and 9-B-10 with the *SDGMS* genotype, were obtained, and their nucleotide sequences were determined using PCR and sequencing (Fig. 1a). We analyzed the sequence variation in this region and identified a 67.6-kb deletion in 62-H-5 relative to 9-B-10 (Fig. 1b). Sequence comparison of 9-B-10 with the reference genomes of NIP and ZS97 showed that 9-B-10 has an almost identical sequence to the ZS97 genome in this region except a 1978-bp insertion upstream of a predicted gene (hereafter referred to as *sdgms*) (Fig. 1b). The NIP genome was 27.7-kb shorter relative to ZS97 in this region containing the same predicted gene without the 1978-kb insertion. An analysis of 330 varieties, including 171 *indica* accessions, 43 *Aus* accessions, 89 *japonica* accessions and 27 other types, using a molecular marker, showed that none of the rice varieties had the 1978-bp DNA insertion (Supplementary Table S2).

To determine whether the 1978-bp DNA insertion is the cause of the dominant genic male sterility, a transformation construct *SDGMS*-N of the *sdgms* gene driven by its native promoter was prepared by PCR amplification of an 8677-bp genomic DNA fragment containing the 1978-bp insertion and 2170-bp upstream region, the 2294 bp gene body (exons and introns) and the 2140-bp 3’region (Supplementary Fig. S3a). The construct was introduced into the NIP variety, producing 25 independent T_0_ plants. All 17 T_0_ transgene-positive plants showed very low spikelet fertility (average 13.5%), whereas spikelet fertility of the 8 transgene-negative plants was much higher (average 65.3%) (Supplementary Table S3). Analysis of two independent T_1_ families from seeds of the transgene-positive T_0_ plants showed that the negative segregants had nearly normal spikelet fertility (70.6% and 64.9%), while very low fertility was observed in the positive segregants (9.7% and 9.6%) (Fig. 1c and Supplementary Table S3). Two independent T_0_ plants that were completely male sterile were used as the female parents to cross with the wild-type parent NIP to produce BC_1_ plants. The average spikelet fertility of the transgene-positive plants was 2.9% and 5.7%, respectively, whereas the negative segregants produced fertility of 70.7% and 62.1% (Supplementary Table S3). This result suggested that the introduced fragment containing the promoter sequence with the 1978-bp insertion and *sdgms* gene caused the dominant male sterility phenotype.

We next constructed *SDGMS*-OE, which contains the 7377-bp genomic DNA fragment with the 1978-bp insertion and the *SDGMS/sdgms* gene driven by the maize ubiquitin promoter, into the NIP variety (Supplementary Fig. S3a). All 26 positive T_0_ plants showed complete male sterility, producing no pollen in the anthers (Fig. 1d and Supplementary Table S3). Three independent T_0_ plants, all highly male sterile, were chosen to cross with wild-type NIP. In all the three BC_1_ populations, negative plants exhibited normal spikelet fertility (67.9%, 67.4% and 69.0%), whereas positive plants showed male sterility with zero spikelet fertility (Supplementary Table S3).

Furthermore, we generated knockout mutants of the *SDGMS* gene using CRISPR/Cas9 in ZS97(*SDGMS*) (Supplementary Fig. S3b). Three *SDGMS^ko^* T_0_ plants with the deletions in the start codon (ATG) of the *SDGMS* gene recovered the fertility (67.4%, 71.7%, 62.3%), whereas the mutations upstream of the start codon of the *SDGMS* gene did not affect fertility (Fig. 1e and Supplementary Table S3). Three fertile T_0_ plants were chosen to observe the spikelet fertility of T_1_ families, and there was no significant difference in fertility between *SDGMS^ko^* and *sdgms* segregants (Supplementary Table S3). We also crossed the sterile T_0_ plants with the wild-type parent ZS97, the fertility of the resulting BC_1_ plants segregated as fertile (82.8%) and sterile (0.6%) groups at a 1:1 ratio (Supplementary Table S3).

We also prepared a *sdgms*-OE construct, which contains the *sdgms* gene body driven by the ubiquitin promoter, and introduced into the NIP variety (Supplementary Fig. S3a). Although enhanced expression of the *sdgms* gene was detected in the transgenic plants (Supplementary Fig. S4), no significant fertility reduction of the transgene-positive plants was observed relative to the negative plants (Supplementary Table S3). We compared the transcript levels of *SDGMS/sdgms* in the spikelets of *SDGMS*-OE and *sdgms*-OE plants at the meiotic stage. The relative expression level of *SDGMS/sdgms* in independent T_1_ lines of *SDGMS*-OE was much higher than that of *sdgms*-OE plants (Supplementary Fig. S4), suggesting that ubiquitin promoter without the 1987-bp insertion could not drive the *sdgms* to an adequate level to produce male sterility.

Taken together, the transformation results suggested that the whole complement, including the *SDGMS/sdgms* coding sequence, the 1978-bp insert and the full-length promoter (or the genomic location), is necessary for SDGMS in its native setting. Less than optimal length of the promoter (or not the right genomic location) would produce less than complete male sterility, like the case of *SDGMS*-N. Ubiquitin promoter could partly compensate for the insufficiency in the promoter leading to complete sterility (*SDGMS*-OE); but without the 1978-bp insert (*sdgms*-OE) it is not sufficient to produce male sterility presumably because of inability to achieve the peak expression level in the specific tissue required for male sterility.

### The 1978-bp DNA insertion activates *SDGMS* expression

To explore the origin and function of the 1978-bp DNA insertion, we conducted a BLAST search in NCBI using the insertion sequence. The results showed that homologous sequences exist widely in different rice genomes, and the best hit (1973-bp, identity 100%) was located on chromosome 2 of ZS97. In view of the pedigree of Sanming-dominant genic male sterility in which ZS97 was one of the parents, the 1978-bp DNA insertion may have been derived from ZS97 (Supplementary Fig. S1a). Using this 1978-bp DNA fragment as the query to search the giriREPBASE database (https://www.girinst.org), it displays the typical structure of the long terminal-repeat (LTR) retrotransposon, which contains two identical LTR sequences (497-bp each) and two identical target site duplications (5-bp each, CTTCT) (Fig. 1b).

Based on rapid amplification of the cDNA ends (RACE), the coding sequences and 3’UTRs of *SDGMS* from 938(*SDGMS*) and *sdgms* from ZS97 were identical, and the 5’UTRs were 239-bp and 368-bp upstream of the start codon (ATG), respectively. The 1978-bp retrotransposon is inserted 94-bp upstream of the start codon of *SDGMS* and thus does not change its protein coding sequence (Supplementary Fig. S3c).

**Figure 2.**
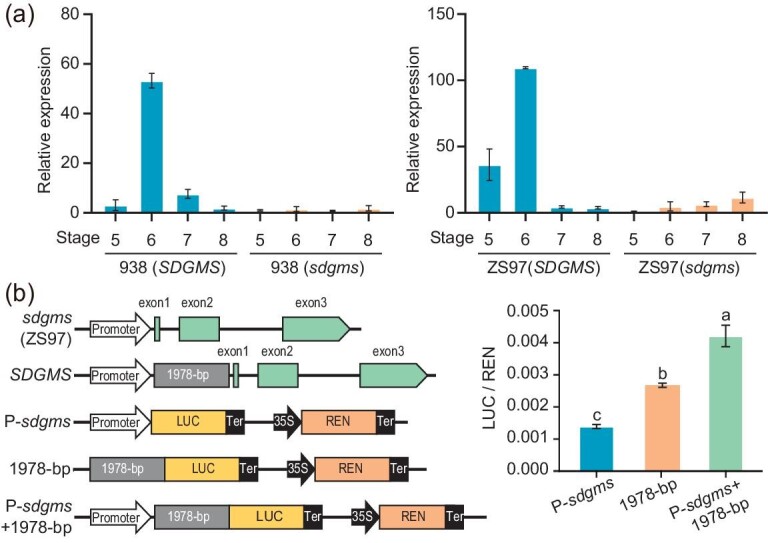
Upregulated expression of *SDGMS* by insertion of a 1978-bp retrotransposon. (a) Relative expression levels of *SDGMS* in young panicles and spikelets of 938(*SDGMS*) and 938(*sdgms*) (left) and ZS97(*SDGMS*) and ZS97(*sdgms*) (right). Stage 5, young panicles at the microspore mother cell stage. Stage 6, spikelet at meiotic stage. Stage 7, spikelet at microspore stage. Stage 8, spikelets at the mature pollen stage. (b) Left, diagrams of reporter vectors containing the upstream sequence of the *sdgms* or *SDGMS* gene using the dual luciferase assay. Right, dual luciferase assay. Different letters indicate significant differences ranked by the LSD test (*P* < 0.01).

To investigate the expression profile of *SDGMS/sdgms*, we searched the plant public RNA-seq Database (http://ipf.sustech.edu.cn/pub/plantrna/?lngdjecbaiecjecj) [22]. *SDGMS/sdgms* was not expressed in most tissues of diverse varieties and barely detectable only in young panicles and spikelets of some varieties (Supplementary Fig. S5). We also analyzed the transcript levels of *SDGMS/sdgms* in various tissues of the NILs. The transcript was not detectable in vegetative tissues such as shoots of 4-leaf stage seedlings, leaves at the tillering stage, and palea and lemma before flowering. The expression level was very low in the young panicle and spikelet of fertile plants 938(*sdgms*) and ZS97(*sdgms*), but became abundant in the young panicle (stage 5) and spikelet (stages 6–7) of sterile plants 938(*SDGMS*) and ZS97(*SDGMS*) (Fig. 2a). RNA *in situ* hybridization revealed that *SDGMS* was specifically expressed in the tapetum of anthers from ZS97(*SDGMS*) at the EM stage, whereas no obvious signal was detected in ZS97(*sdgms*) anthers at this stage (Supplementary Fig. S6). This expression pattern is in accordance with the strong abnormal PCD signal detected in 938(*SDGMS*) at the EM stage, which is also supported by the real-time PCR and RNA-seq data showing that *SDGMS* is highly expressed at the meiotic stage. These results indicate that the dominant male sterility resulted from the acquired expression of *SDGMS*; the insertion of the 1978-bp retrotransposon boosts expression of *SDGMS* in the tapetum cells during male gamete development resulting in male sterility.

We used the dual luciferase reporter system to assess the effect of the 1978-bp retrotransposon on gene expression. The native promoter of the *sdgms* gene (2170-bp upstream sequence of *sdgms*), the native promoter of *SDGMS* containing the retrotransposon (2170-bp upstream sequence plus 1978-bp retrotransposon), and the retrotransposon alone (1978-bp) were used to drive the expression of the coding sequence of firefly luciferase in rice protoplasts (Fig. 2b). The results showed that the 1978-bp retrotransposon alone produced significantly higher firefly luciferase activity than the native promoter of *sdgms*, and the native promoter of *SDGMS* containing the 1978-bp retrotransposon could further increase the activity of firefly luciferase (Fig. 2b). Thus, the 1978-bp retrotransposon could both prime and enhance the expression of *SDGMS*.

### The *SDGMS*/*sdgms* gene encodes a ribosome inactivating protein

The predicted SDGMS/sdgms protein was 285 amino acids in length and annotated as a ribosome inactivating protein (IPR110574) by InterPro (http://www.ebi.ac.uk/interpro/). No signal peptide was identified by SignalP 5.0 (https://services.healthtech.dtu.dk/service.php?SignalP-5.0).

**Figure 3.**
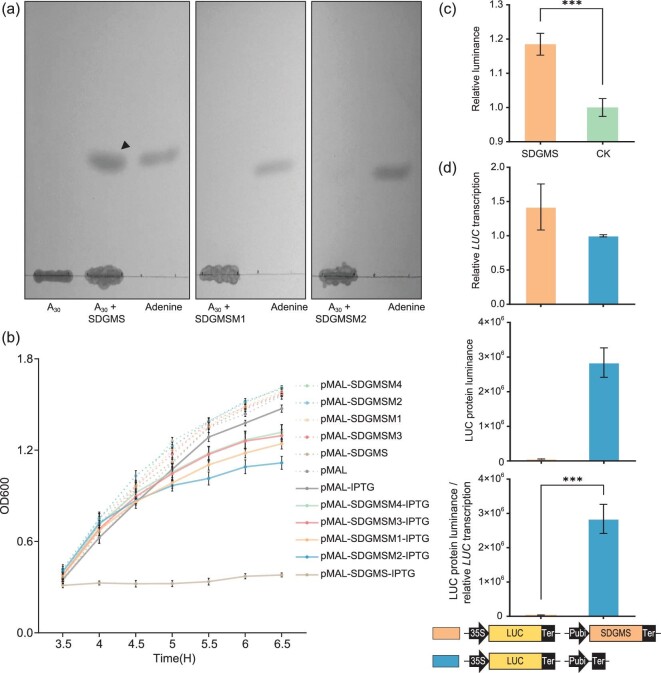
N-glycosidase activity of SDGMS and catalytic site-mutated SDGMS protein. (a) A_30_-ssDNA is incubated with SDGMS, SDGMSM1 and SDGMSM2 proteins at 37°C for 8 hours and is separated by thin layer chromatography. The arrow indicates the released adenine. SDGMSM1 (Tyr100 to alanine) and SDGMSM2 (Glu198 to alanine) are catalytic site-mutated forms of SDGMS. (b) Influence of SDGMS and catalytic site-mutated SDGMS expression on *E. coli* growth. Induced expression of the SDGMS protein hinders the growth of *E. coli* and is cytotoxic to bacteria, while the mutated form of SDGMS has no effect on the growth of *E. coli.* SDGMSM3 (Arg201 to alanine) and SDGMSM4 (Phe236 to alanine) are catalytic site-mutated forms of SDGMS. IPTG indicates induced protein expression by IPTG. (c) Rice total RNA was incubated with or without (CK) SDGMS protein at 37°C for 60 min, and the concentration of released adenine was detected by fluorescence. (d) SDGMS protein inhibits LUC protein translation but not transcription in rice protoplasts. The two constructs were co-transformed to rice protoplast and the transcription and translation products were measured after 16 h incubation. Upper, relative transcription level of *LUC*. Middle, luminance of LUC protein. Lower, the ratio of luminance of LUC protein to relative transcription level of *LUC.* The illustrations under the histograms indicate the vectors used in rice protoplast transformation.

Ribosome inactivating proteins are classified as RNA N-glycosidases that catalyze the depurination of adenine in the conserved α-sarcin/ricin loop (α-SRL) of the 28S/25S/23S rRNA [23,24]. To assess whether *SDGMS/sdgms* encodes a ribosome inactivating protein, we obtained SDGMS/sdgms protein by expressing its coding sequence in *Escherichia coli*. An *in vitro* protease assay showed that it depurinated and cleaved the N-glycosidic bond of A30-ssDNA, which mimicked the α-sarcin/ricin loop, and released adenine (Fig. 3a). Expression of SDGMS/sdgms protein in *E. coli* induced by isopropyl-β-D-thiogalactopyranoside (IPTG) impeded *E. coli* growth (Fig. 3b). The SDGMS/sdgms protein exhibited N-glycosidase activity on RNA as evidenced by the released adenine after incubation with rice total RNA (Fig. 3c); SDGMS/sdgms inhibited protein translation *in vivo*, as indicated by its inhibition of luciferase expression at the translation level but not at the transcription level in rice protoplasts (Fig. 3d). We further investigated the key catalytic residues of SDGMS by mutating the four RIP conserved residues (Tyr100, Glu198, Arg201 and Phe236) of SDGMS to alanine. Expressing each of the four catalytic site-mutated variants of SDGMS had no effect on the growth of *E. coli* (Fig. 3b), and the mutated form of Tyr100 (SDGMSM1) and Glu198 (SDGMSM2) failed to depurinate and cut the N-glycosidic bond of an A_30_-ssDNA (Fig. 3a). These results demonstrated that the *SDGMS/sdgms***-**encoded protein possesses N-glycosidase activity and is indeed a ribosome-inactivating protein, and the catalytic residues are necessary for N-glycosidase activity and cytotoxicity to *E. coli*.

### *SDGMS* activates defence response in anther disrupting pollen development

To investigate the possible mechanism of male sterility caused by *SDGMS*, we collected young panicles of 938(*SDGMS*) and 938(*sdgms*) at MMC (microspore mother cell) and spikelets at EM (early meiotic), LM (late meiotic) and MP (mature pollen) stages, and identified differentially expressed genes (DEGs) using RNA-Seq transcriptome analysis. A total of 22, 236, 3318 and 4694 DEGs were detected at MMC, EM, LM and MP, respectively, of which 14, 177, 1008 and 686 genes were upregulated at these stages, and 8, 59, 2310 and 4008 genes were downregulated (Supplementary Fig. S7a and Supplementary Table S4).

We checked the key genes regulating tapetal PCD as reported in rice [25]. The expression of *UDT1, OsGAMYB, bHLH142, TDR, EAT1, PTC1, DTC1, OsC6, OsAP25, OsAP37* and *OsCP1* was downregulated in 938(*SDGMS*) at LM, during which the fertile anther tapetal cells undergo intense PCD (Supplementary Fig. S7b). This was consistent with the results of the TUNEL assay showing abnormal PCD of tapetal cells of *SDGMS* anthers.

We performed Gene Ontology (GO) enrichment analyses to classify the DEGs at the EM and LM stages. GO analysis of upregulated genes at EM revealed that GO terms related to regulation of gene expression and translation were significantly enriched [false discovery rate (FDR) < 0.05], such as gene expression (GO:0 010 467), amino acid activation (GO:0 043 038) and tRNA aminoacylation for protein translation (GO:0 006 418), which may be caused by the protein translation inhibition of SDGMS. Response to biotic stimulus (GO:0 009 607) was also enriched at EM. No GO term was enriched in downregulated genes at EM (Supplementary Table S5).

At LM, GO analysis indicated that upregulated genes were enriched for 53 biological processes, including regulation of gene expression (GO:0 010 468), posttranslational protein modification (GO:0 043 687), response to stimulus (GO:0 050 896) and programmed cell death (GO:0 012 501) (Supplementary Fig. S7c and Supplementary Table S5). GO terms associated with the ubiquitin-dependent protein catabolic process (GO:0 006 511) and lipid metabolic process (GO:0 006 629) were enriched in downregulated genes at LM (Supplementary Fig. S7c and Supplementary Table S5).

In particular, among the upregulated DEGs, all the 30 genes in the GO term programmed cell death (GO:0 012 501) were annotated as NB-ARC or NB-LRR. The GO term protein amino acid phosphorylation (GO:0 006 468) included 108 genes, 48 associated with receptor-like protein kinase were enriched. Of the 84 genes in the GO term transcription regulation (GO:0 045 449), 21 were *WRKY*s (Supplementary Table S6). Furthermore, 20 and 23 pathogenesis-related (*PR*) genes (total 113 in the rice genome) were upregulated at EM and LM, respectively (Supplementary Table S7).

**Figure 4.**
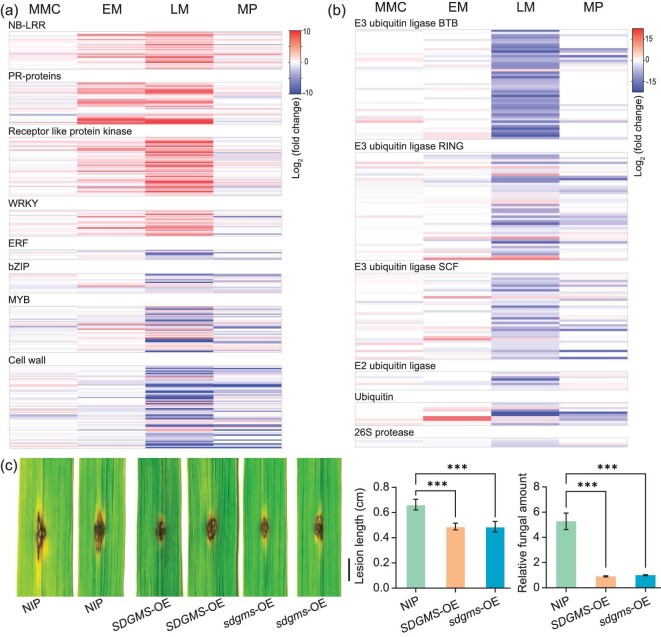
*SDGMS* activates defence response in anther. (a) Differentially expressed genes related to biotic stress. Each row indicates a differentially expressed gene. The colour of the row represents the expression level of differentially expressed genes based on the log_2_-fold change. Red rows represent upregulation and blue rows represent downregulation. (b) Differentially expressed genes related to protein degradation. (c) Disease symptoms, lesion length and relative fungal amount of NIP, *SDGMS*-OE and *sdgms*-OE leaves after inoculation with *M. oryzae* RB22. Scale bar = 0.5 cm.

MAPMAN analysis of DEGs at LM obtained similar results to the GO analysis. Genes related to biotic stress, including *R* genes, *PR* genes and *WRKYs*, were upregulated; and conversely, genes involved in ubiquitin-dependent degradation were downregulated (Fig. 4a and b and Supplementary Table S8). Thus, both the MAPMAN and GO results showed that transcription was reprogrammed in 938(*SDGMS*) spikelets relative to 938(*sdgms*); and the genes related to response to biotic stress were induced.

*NLR*s play a key role in recognizing pathogen effectors and activating effector-triggered immunity (ETI); however, overaccumulated *NLR*s could be harmful to plant growth and development [26–30]. E3 ubiquitin ligases are reported to suppress the *NLR*-induced immune response to avoid autoimmunity [31,32], downregulation of the genes related to ubiquitin-dependent degradation may hamper this activity. Therefore, we hypothesized that the ribosome-inactivating protein SDGMS/sdgms may have the function for biotic stress response [24,33]. To test this hypothesis, we inoculated leaves of NIP, *SDGMS*-OE and *sdgms*-OE plants with blast fungus at the tillering stage. The results showed that overexpression of *SDGMS*/*sdgms* in rice could enhance resistance to *M. oryzae* RB22 as measured by the length of disease lesions and the relative amount of fungal pathogen (Fig. 4c). Therefore, we speculate that the expression of *SDGMS* specifically activates the defence pathway in anthers and triggers the hypersensitive response in tapetal cells.

## DISCUSSION

**Figure 5.**
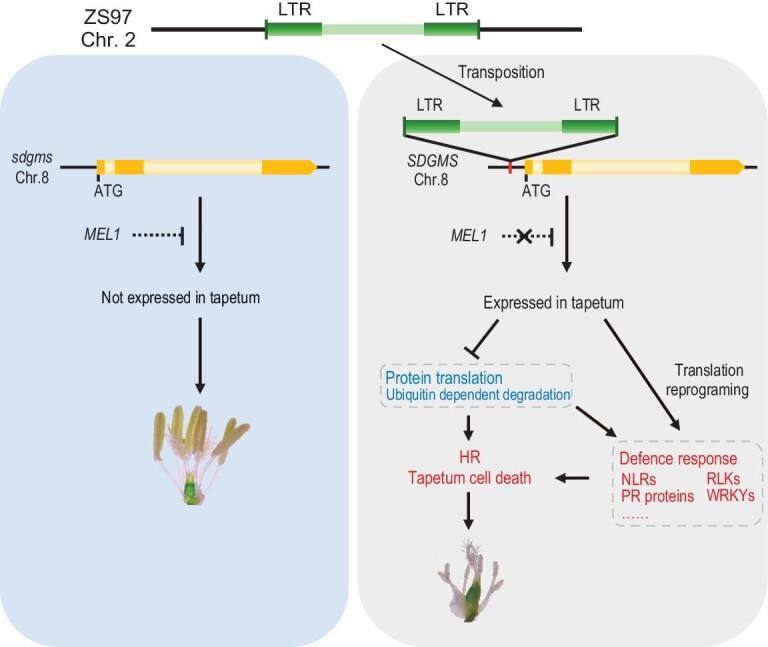
A working model of dominant male sterility caused by *SDGMS.* The red colour indicates an upregulation response involved in the sterility process, and blue indicates a downregulation response.

Based on the results of our work, we propose a model for the origin and function of the SDGMS. A 1978-bp LTR retrotransposon was accidentally activated and transposed from the genomic region on chromosome 2 of ZS97 to the promoter region of *SDGMS* during the breeding process, which activated the expression of *SDGMS* in anther by serving both as a primer and enhancer (Fig. 5). *SDGMS/sdgms* encodes a ribosome inactivating protein having N-glycosidase activity on RNA and thus may result in irreversible modification of the target A residue, which blocks the activity of elongation factor (EF)-1- and EF-2-dependent GTPase and renders the ribosome unable to bind EF-2, thereby repressing translation [23,24,34,35]. This process consequently causes endogenous biotic stress, thus triggering the hypersensitive response, leading to abnormal PCD of tapetal cells resulting in abortion of the anther locule.

An interesting observation was that expression of *sdgms* was lightly upregulated in the anther of a sterile *mel1* mutant, a mutant of a germ cell-specific AGO protein MEL1 that causes irregularly sized, multinucleated, and vacuolated pollen mother cells in developing anthers via 21-nt phasiRNA-mediated gene silencing [36–39]. By comparison, the expression of *sdgms* remained very low in other male sterility mutants, such as *eat1, msp1, ostdl1, dcl3b, osmyb80* and *ago18* (Supplementary Fig. S8). Therefore, it is likely that *MEL1* could suppress the expression level of *sdgms* to ensure the normal development of anther, whereas the insertion of the 1978-bp retrotransposon altered the transcription of *SDGMS* that could have been suppressed by *MEL1*.

The demonstration of an LTR retrotransposon insertion in the promoter region of a ribosome-inactivation protein giving birth to a gene for stable dominant male sterility in rice provides a fresh example that TE movement is an ongoing process in genome evolution. Such a process can create novel traits with practical utility contributing to genic diversity and phenotypic novelty.

The SDGMS rice provides a very powerful tool to facilitate outcrossing, which can be explored in many ways for diverse breeding programs. This includes, but is not limited to: (1) precise introgressing of a desired allele of a gene for an agronomic trait by successive backcrosses for rapid directional improvement of elite cultivars [40]; (2) random introgressing of genomic segments from doner lines by backcrosses to produce near isogenic introgressed lines (NIILs) to broaden the genetic basis of the breeding parents [41]; (3) recurrent selection for cyclic population improvement involving a large number of parental lines in a single breeding program, which may simultaneously improve multiple traits, such as better nutrition use efficiency, higher resistance to stress, and high yield potential and quality. Moreover, using any of the male sterile lines presented in this study as the starting point, dominant male sterile lines can be developed for any breeding parents thus eliminating the need for hand emasculation, which will fundamentally improve the efficiency of breeding programs.

## MATERIALS AND METHODS

Detailed materials and methods are available in the supplementary information.

## Supplementary Material

nwad210_Supplemental_Files
