# Supplementary material for: Spontaneous movement of a retrotransposon generated genic dominant male sterility providing a useful tool for rice breeding
Source: Natl Sci Rev. 2023 Aug 7;10(9):nwad210. doi: 10.1093/nsr/nwad210 (PMC10446136; doi:10.1093/nsr/nwad210)
Supplement: nwad210_Supplemental_Files [file nwad210_supplemental_files.zip › 2023-440 Supplementary information.docx]

**Supplementary information for**

**Spontaneous movement of a retrotransposon generated genic dominant male sterility providing a useful tool for rice breeding**

Conghao Xu^1^, Yifeng Xu^2^, Zhengji Wang^1^, Xiaoyu Zhang^1^, Yuying Wu^1^, Xinyan Lu^1^, Hongwei Sun^1^, Lei Wang^1^, Qinglu Zhang^1^, Qinghua Zhang^1^, Xianghua Li^1^, Jinghua Xiao^1^, Xu Li^1^, Mingfu Zhao^3^, Yidan Ouyang^1,*^, Xian-Bo Huang^4,*^, Qifa Zhang^1,*^

^1^National Key Laboratory of Crop Genetic Improvement and National Centre of Plant Gene Research (Wuhan), Hubei Hongshan Laboratory, Huazhong Agricultural University, Wuhan 430070, China.

^2^Ningde Inspection and Testing Centre for Agricultural Product Quality and Safety, Ningde 352100, China

^3^Fujian Academy of Agricultural Sciences, Fuzhou 350018, China

^4^Sanming Institute of Agricultural Sciences, Shaxian 365509, Fujian, China

^*^To whom correspondence should be addressed.

Email: diana1983941@mail.hzau.edu.cn; huangxb_2000@163.com; qifazh@mail.hzau.edu.cn.

**This PDF file includes:**

Supplemental materials and methods

Figures S1 to S8

Tables S1 to S9

Supplemental references

**SUPPLEMENTAL MATERIALS AND METHODS**

**Plant growth and fertility investigation**

All plants used in this study were planted and investigated in the experimental field of Huazhong Agriculture University, Wuhan, China. Anthers of mature flowers were sampled for pollen fertility using the I_2_-KI staining method. Mature panicles were harvested to examine spikelet fertility.

**Fine-mapping of *SDGMS***

A BC_7_F_1_ segregation population for mapping *SDGMS* was generated from successive backcrosses between 938(*SDGMS*) and 938, using the sterile progeny as the female parent. Molecular markers used for mapping are listed in Supplementary Table S9.

**Construction of a BAC library**

To construct a bacterial artificial chromosome (BAC) library, the genomic DNA of 938 (*SDGMS*) was partially digested with *Hin*dIII, and the purified fragments were inserted into the vector pIndigoBAC536-S [1]. Two BAC clones, 62-H-5 and 9-B-10, were screened and genotyped by PCR and sequencing. Sequencing of 62-H-5 and 9-B-10 was performed using Illumina HiSeq 4000 and PacBio Sequel Sequencer at Nextomics Biosciences (Wuhan) Co., Ltd.

**Determination of the full-length transcripts**

Young spikelets of 938(*SDGMS*) and ZS97 at the meiosis stage were collected from the field and stored in liquid nitrogen. Total RNA was extracted using an RNA extraction kit (Invitrogen) following the manufacturer’s instructions.

To synthesize the first-strand cDNA, 1 μg total RNA was reverse-transcribed according to the protocol provided by the SMARTer® RACE 5’/3’ Kit (Takara), with a total volume of 100 μl.

For 5’RACE, two rounds of nested PCR amplification were performed using two nested universal primers (UPM-L and UPM-S) provided by the kit and gene-specific primers (RACE-5-L paired with UPM-L for the first round and RACE-5-S paired with UPM-S for the second round, Supplementary Table S9). The product from the first round was diluted and used as the template. For 3’RACE, the gene-specific primer RACE-3-L was used for the first round of amplification paired with UPM-L, and RACE-3-S was used for the second round of reaction paired with UPM-S. The candidate products were purified and cloned into pGEM-T (Promega) for sequencing.

**Expression analysis**

Total RNA was isolated with TRIzol reagent (Invitrogen) as described above. cDNA was reverse-transcribed using the HiScript III 1st Strand cDNA Synthesis kit (Vazyme, China). Real-time quantitative PCR (qRT‒PCR) was performed using the SYBR Green I Master PCR kit on the ABI Vii7. For qRT‒PCR analysis of *SDGMS/sdgms*, RNA from young panicles at the microspore mother cell stage and spikelets at the meiotic stage, microspore stage, and mature pollen stage was prepared and reverse-transcribed to cDNA. Samples of each stage were biologically repeated three times, with three technological replicates each. The rice *ubiquitin* gene was used as an internal control for normalization. The relative expression levels were measured using the 2^−ΔΔCt^ analysis method [2]. For RT‒PCR analysis of *SDGMS* and *ubiquitin*, the RNA from the shoot at the 4-leaf stage, leaves at the tillering stage and palea and lemma at the mature pollen stage was prepared and reverse-transcribed to cDNA, with the PCR program setting for 29 cycles. All primers used for gene expression analysis are listed in Supplementary Table S9.

**Expression and purification of SDGMS in *E. coli***

The coding sequence of *SDGMS* and its four site-mutated forms were amplified and cloned into the pMAL-c2x vector (Biolabs) to obtain the pMAL-*SDGMS*, pMAL-*SDGMSM1* (Tyr100 to alanine), pMAL-*SDGMSM2* (Glu198 to alanine), pMAL-*SDGMSM3* (Arg201 to alanine) and pMAL-*SDGMSM4* (Phe236 to alanine) constructs. These constructs were introduced into *E. coli* strain DE3 and cultured at 37°C. When the A600 reached 0.6, the SDGMS protein was expressed by adding isopropyl 1-thio-D-galactopyranoside at a final concentration of 0.5 mM. After 8 h, the cells were collected by centrifugation and resuspended in TPS buffer [Tris-HCl (20 mM), NaCl (200 mM), EDTA (1 mM pH 7.0)]. The SDGMS protein was purified based on the pMAL Protein Fusion and Purification Instruction Manual (Biolabs) and was concentrated by ultrafiltration and freeze drying. All primers used for protein expression are listed in Supplementary Table S9.

**Adenine-releasing assay by SDGMS and adenine assay**

Adenine-releasing assay was performed as previously reported [3]. A total of 20 µg A30-ssDNA (5’-AAAAAAAAAAAAAAAAAAAAAAAAAAAAAAAA-3’) was incubated with 20 µg SDGMS, SDGMSM1 and SDGMSM2 protein in a 10 µl reaction mixture at 37℃ for 8h respectively. The mixture was separated by thin-layer chromatography on silica gel F254 plates (Sangon Biotiec-h, Shanghai, China) with chromatographic solution consisting of acetonitrile (18) : water (6) : ammonia (0.6). After drying at room temperature, the spots were visualized by UV (254 nm). Adenine (Sangon Biotech, Shanghai, China) was used as a control.

Adenine was quantified using the method described by [4]. A total of 5 µg of total RNA was incubated with 20 µg of SDGMS protein in a 50 µl volume system containing Tris–HCl (250 mM, pH 7.6), KCl (25 mM) and MgCl_2_ (5 mM). After incubation at 37°C for 1 h, the total RNA was precipitated with ethanol. The supernatant was diluted with water to 1 ml after centrifugation, and 0.4 ml chloroacetaldehyde (0.14 M) with sodium acetate (100 mM, pH = 5) was added to each sample. The sample was incubated at 85°C for 1 h. Fluorescence measurements were performed at an excitation wavelength of 310 nm and an emission wavelength of 410 nm after cooling to room temperature.

**Translation inhibition assay**

The coding sequence of *SDGMS* and firefly luminance gene (LUC) driven by the CaMV 35S promoter was cloned into the pCambia1300 vector and co-transformed into rice protoplasts, with the empty vector as a control. After culture for 16 h at room temperature in the dark, the relative expression level and luciferase activity of LUC were measured. The ratio of luciferase activity and relative expression level was taken as an indicator of translation level.

**Dual luciferase assay**

The dual luciferase assay was performed using the Dual-Luciferase® Reporter Assay System (Promega). The 1978-bp DNA insertion, the native promoter of *SDGMS* (2170-bp), and the native promoter (2170-bp) of *SDGMS* plus the 1978-bp DNA insertion were amplified from the 938(*SDGMS*) and ZS97 genomes and cloned into the pGreenII 0800-LUC vector to drive the expression of *LUC*. The internal reference gene Renilla luciferase gene (*REN*) in the same vector was driven by the CaMV 35S promoter. The constructs were transformed into rice protoplasts and cultured for 16 h at room temperature in the dark. The luminance of LUC and REN was measured using the Tecan Spark™ 10 M microplate reader.

**Traverse sections and TUNEL assays**

The young panicles and spikelets in various stages were collected and fixed with 50% FAA [ethyl alcohol (50%), 38% methanol (10%), acetic acid (5%)]. The sample was embedded in paraffin and cut into sections following the method described previously [5]. Paraffin sections of panicles and spikelets were used for TUNEL assays using the DeadEnd™ Fluorometric TUNEL system (Promega). The section sample was analysed under an ECLIPSE Ni-E microscope (Nikon).

***In situ* hybridization**

A Primer pair SDGMSinsituF and SDGMSinsituR was used to prepare the probes for *SDGMS* (Supplementary Table S9). The sense and antisense probes were transcribed with T7 RNA polymerase and SP6 RNA polymerase (Roch) respectively. The probes were labelled with digoxigenin by DIG RNA Labeling Mix (Roch), and hydrolyzed to 200 nt. Hybridization was performed as previously described [6], and detected by anti-Digoxigenin-AP (Roch).

**RNA sequencing and transcriptomic analyses**

The 1.5 cm young panicles (MMC), 0.45-0.5 mm spikelet (EM), 0.6-0.65 mm spikelet (LM), and spikelet before flowering of 938(*SDGMS*) and 938(*sdgms*) were harvested with two biological replicates. The developmental stages of anthers were determined by paraffin sections of 938(*sdgms*). RNA was extracted using TRIzol reagent (Invitrogen) as described above. Libraries were constructed using 2 μg RNA with the TruSeq RNA Library Preparation Kit v2 (Illumina). The paired-end RNA-seq libraries were sequenced with the Illumina HiSeq xten platform (2×150 bp).

The reads were aligned to the NIP reference genome (MSU version 7.0, http://rice.uga.edu/) using Hisat2 v2.2.0 [7]. The gene counts of each library were calculated by HTSeq v0.11.2 [8]. DEGs were obtained using the DESeq2 v1.36.0 package [9]. GO enrichment analyses were performed using agriGO v2.0 [10]. MapMan (3.6.0RC1) software was downloaded from the MapMan website (mapman.gabipd.org) and was used to analyse the metabolic pathways and biological processes [11].

**Rice blast fungus inoculation**

*Magnaporthe oryzae* RB22 isolates were cultured on oatmeal tomato medium (oatmeal 40 g/L, tomato juice 150 ml/L, agar 20 g/L, pH 7.5) at 25°C for 7 d. Conidia were collected from the medium. Fully expanded leaves of rice plants at tillering stage were harvested and inoculated with an *M. oryzae* spore suspension (2 μl) by the spot inoculation method described previously [12]. The inoculated leaves were placed in a light-moist environment at 25°C for 6 d, and disease symptoms were quantified by measuring the lesion length. Relative fungal amount was measured by DNA-based quantitative PCR (qPCR) using the threshold cycle value (CT) of *M. oryzae* 28S rDNA against the CT of rice genomic *ubiquitin* DNA, primers used are listed in Supplementary Table S9.

**SUPPLEMENTAL FIGURES AND TABLES**


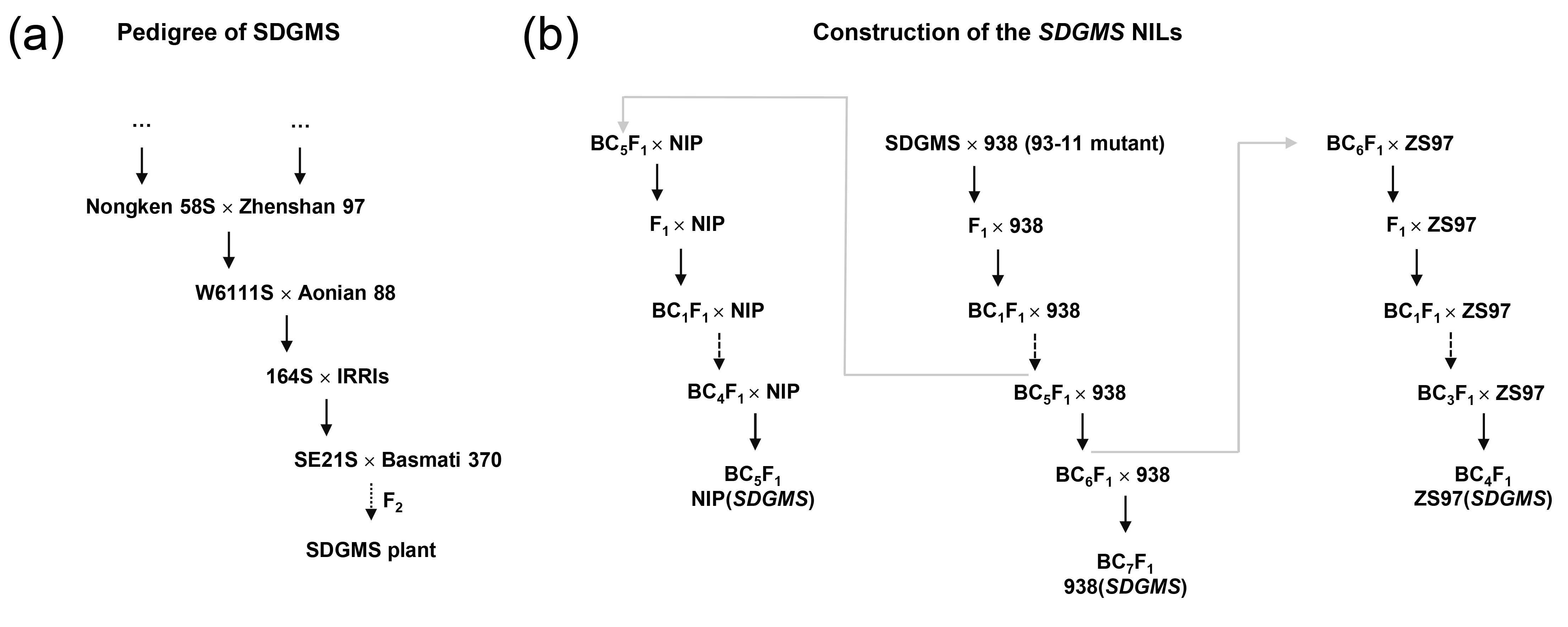


**Figure S1.** Construction of the near isogenic lines (NILs) for SDGMS. (a) The source of the SDGMS germplasm. (b) The construction of the three SDGMS NILs, 938(*SDGMS*), ZS97(*SDGMS*), and NIP(*SDGMS*), which contain chromosomal segments with the *SDGMS* gene (in a heterozygous state) from the SDGMS mutant in the genetic backgrounds of 938 (a mutant from 93-11), Zhenshan 97 (ZS97), and Nipponbare (NIP).


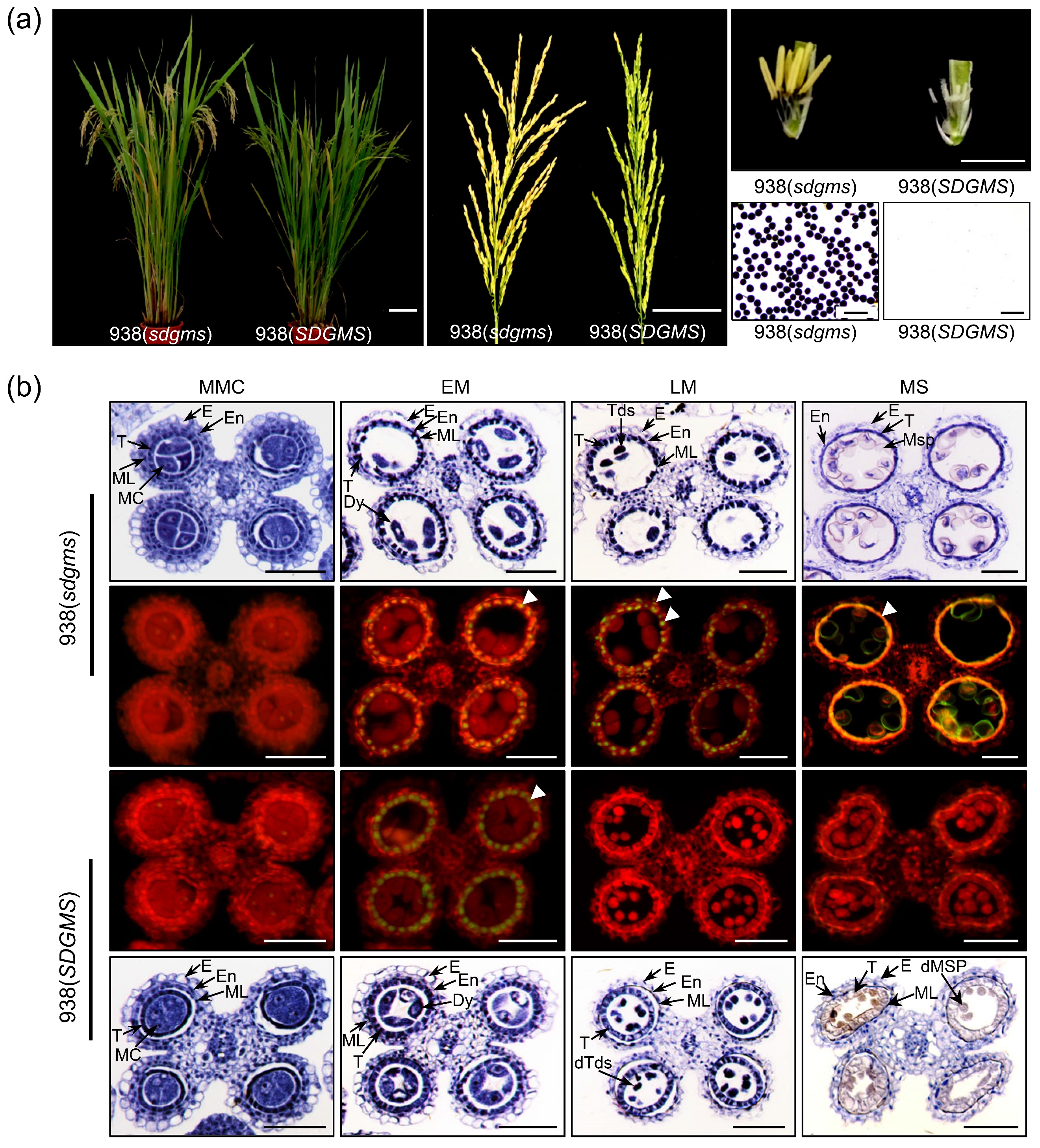


**Figure S2.** Abnormal PCD of tapetal cells in the SDGMS plants. (a) Whole plants, panicles, anthers and pollen grains stained with K_2_-KI of 938(*sdgms*) and 938(*SDGMS*) at the maturity stage. Scale bar for plants = 10 cm, scale bar for panicles = 5 cm, scale bar for anthers = 5 mm, scale bar for pollen grains = 50 μm. (b) TUNEL assays of *SDGMS*. The first and fourth lines, traverse sections of anther locule of 938(*sdgms*) and 938(*SDGMS*), respectively. The second and third lines, TUNEL assays of 938(*sdgms*) anthers and 938(*SDGMS*) anthers, respectively. The red signal is propidium iodide staining, and yellow fluorescence is the merged signal from TUNEL (green) and propidium iodide staining. Scale bar = 50 μm. MMC, microspore mother cell stage. EM, early meiotic stage. LM, late meiotic stage. MS, microspore stage. E, epidermis. En, endothecium. ML, middle layer. T, tapetum. MC, microspore mother cell. Dy. Dyad cell. Tds, tetrads. Msp, microspore. dTds, degraded tetrads. dMSP, degraded microspore.


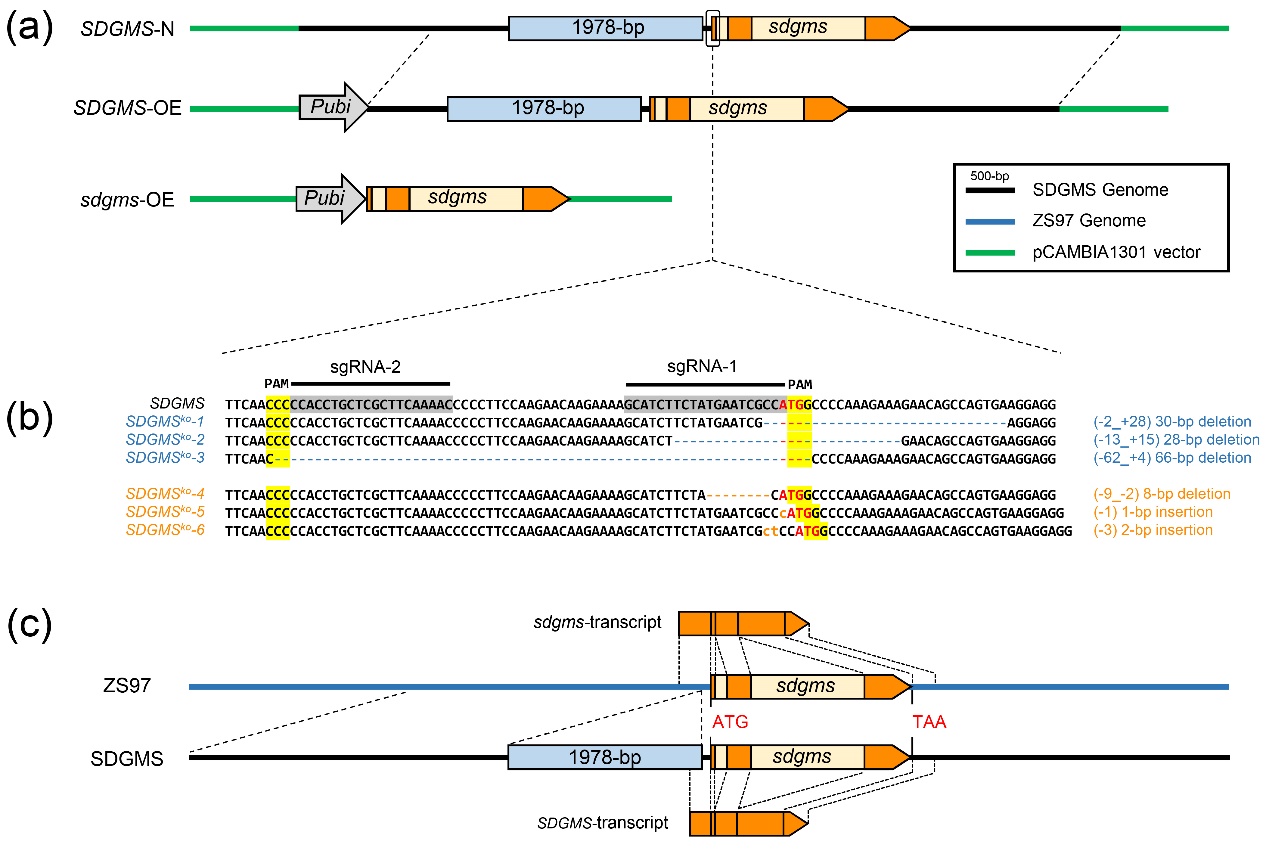


**Figure S3.** Illustration of the constructs used for genetic transformation. (a) Illustration of the *SDGMS*-N, *SDGMS*-OE and *sdgms*-OE constructs. The black line indicates the SDGMS genome, the blue line indicates the ZS97 genome, the green lines indicate the pCAMBIA1301 vector, the grey arrow indicates the maize *ubiquitin* promoter. (b) Gene-editing information of the *SDGMS^ko^* mutants. The dashed lines indicate the deletion, and the orange lowercase letters within the sequence indicate the insertion. The letters in red indicate the start codon of *sgdms*. (c) The full-length transcripts of *sdgms* in ZS97 and *SDGMS* in 938(*SDGMS*) using RACE.


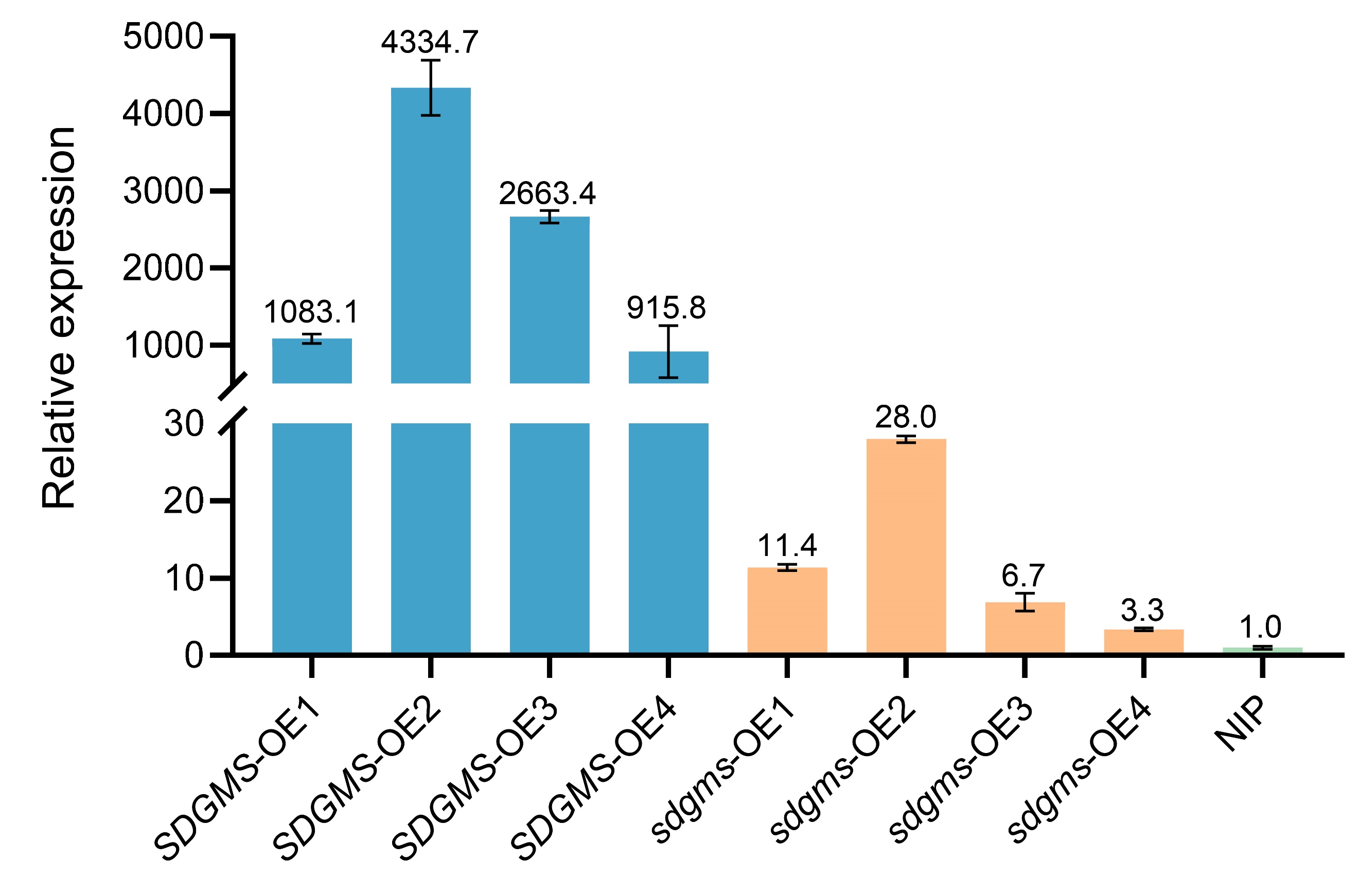


**Figure S4.** Relative expression levels of *SDGMS/sdgms* in spikelets of *SDGMS*-OE, *sdgms*-OE and NIP plants at the meiotic stage. The numbers above the columns indicate the relative expression levels based on data from three biological repeats and the error bars are S.E.M (n=3).


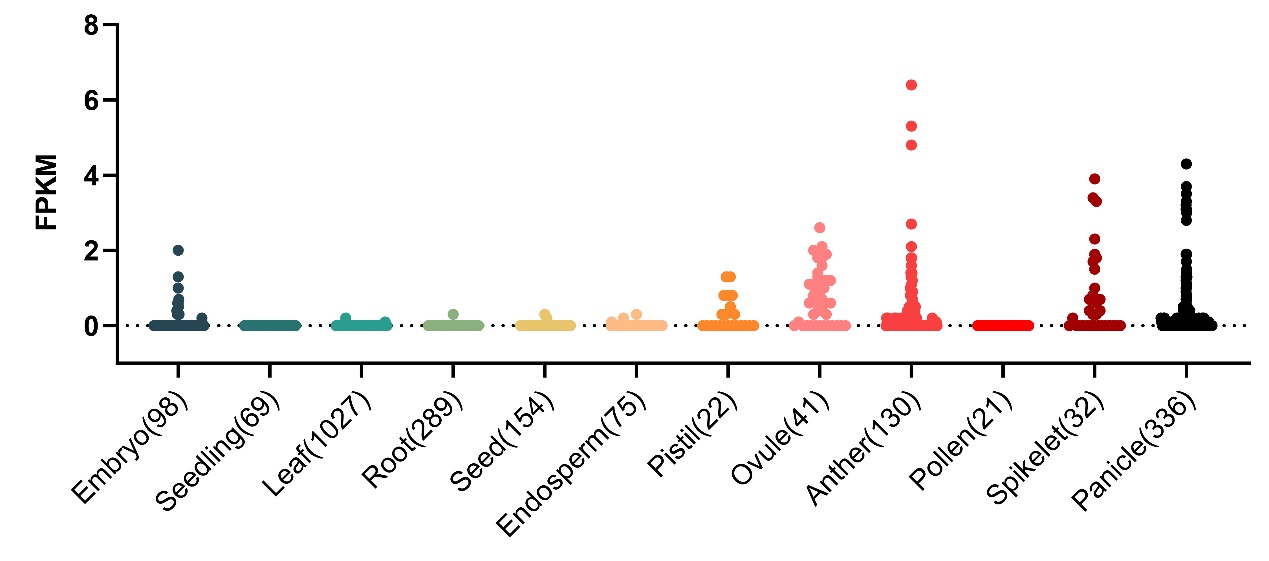


**Figure S5.** The expression profile of *SDGMS/sdgms* in rice tissues. Transcript levels of *SDGMS/sdgms* in a wide range of rice varieties and tissues were obtained from the public RNA-seq databases (<http://ipf.sustech.edu.cn/pub/plantrna/?lngdjecbaiecjecj>). The numbers in parentheses indicate the number of RNA-seq libraries searched.


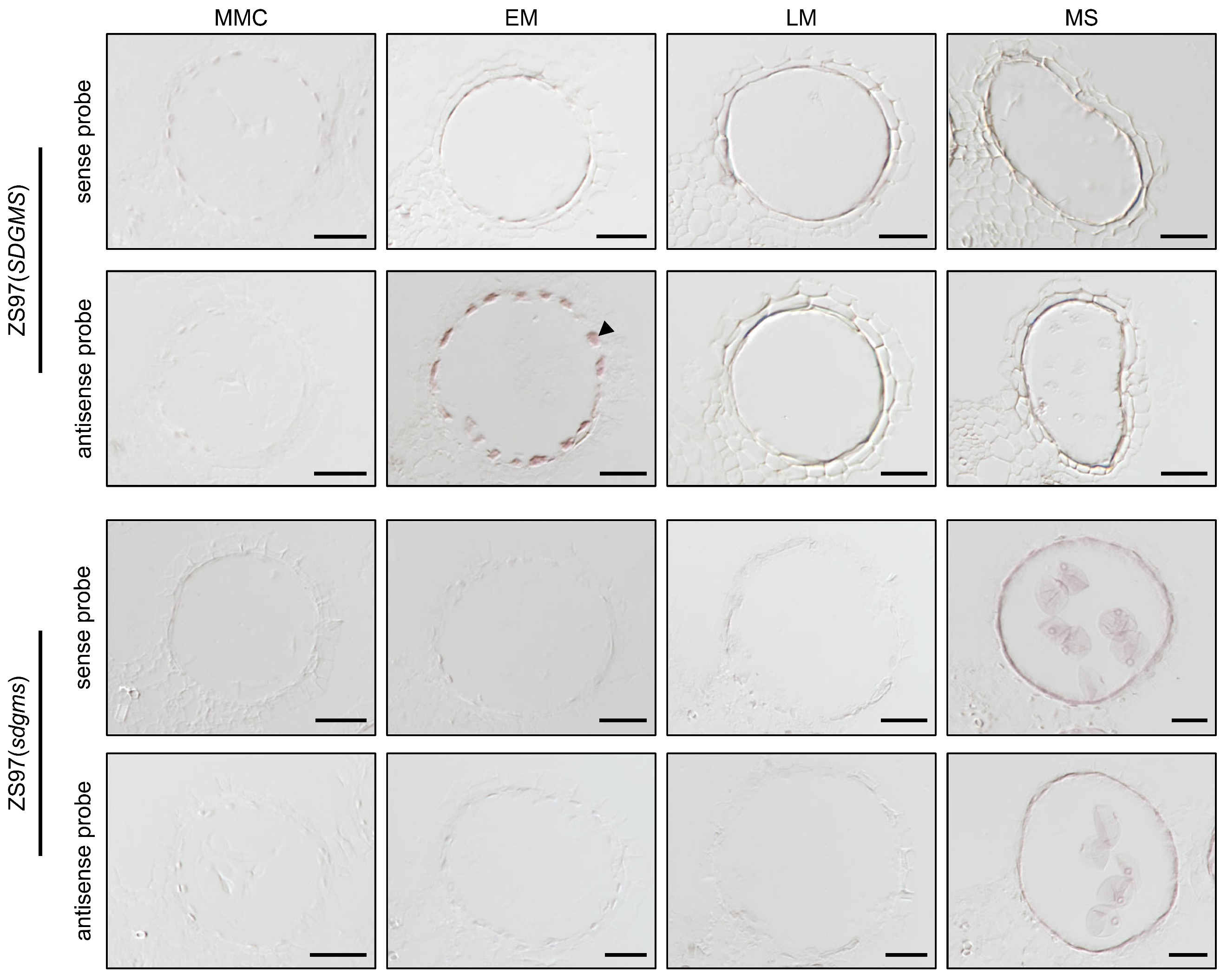


**Figure S6.** RNA *in situ* hybridization of *SDGMS* in ZS97(*SDGMS*) and ZS97(*sdgms*) anthers at different developmental stages. The photos show one of the four lobes of an anther, and the arrow indicates the expression signal of *SDGMS.* Scale bar = 20 μm. MMC, microspore mother cell stage. EM, early meiotic stage. LM, late meiotic stage. MS, microspore stage.

**
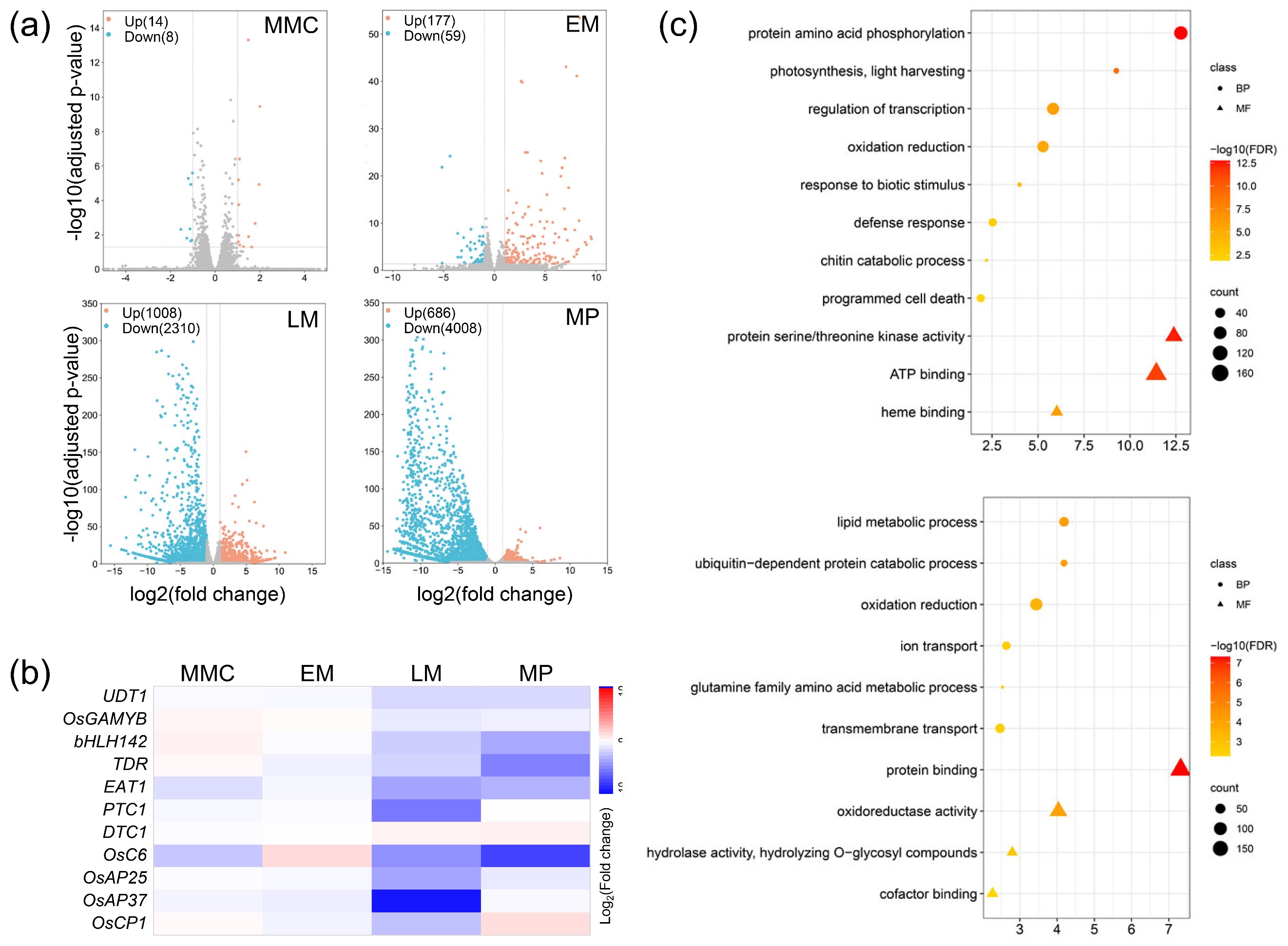
**

**Figure S7.** Differentially expressed genes (DEGs) detected between 938(*SDGMS*) and 938(*sdgms*). (a) Scatter plots of DEGs in different stages with the base 2 logarithm fold change and probability of adjusted p value < 0.05. (b) The heatmap was generated with log_2_(Fold change). Red indicates upregulated expression, and blue indicates downregulated expression. (c) Significantly (FDR < 0.05) enriched GO terms of upregulated genes (top) and downregulated genes (bottom) in 938(*SDGMS)* spikelets at LM. The colour of each dot represents the significance level of enrichment based on the FDR value. MF, molecular function. BP, biological process.


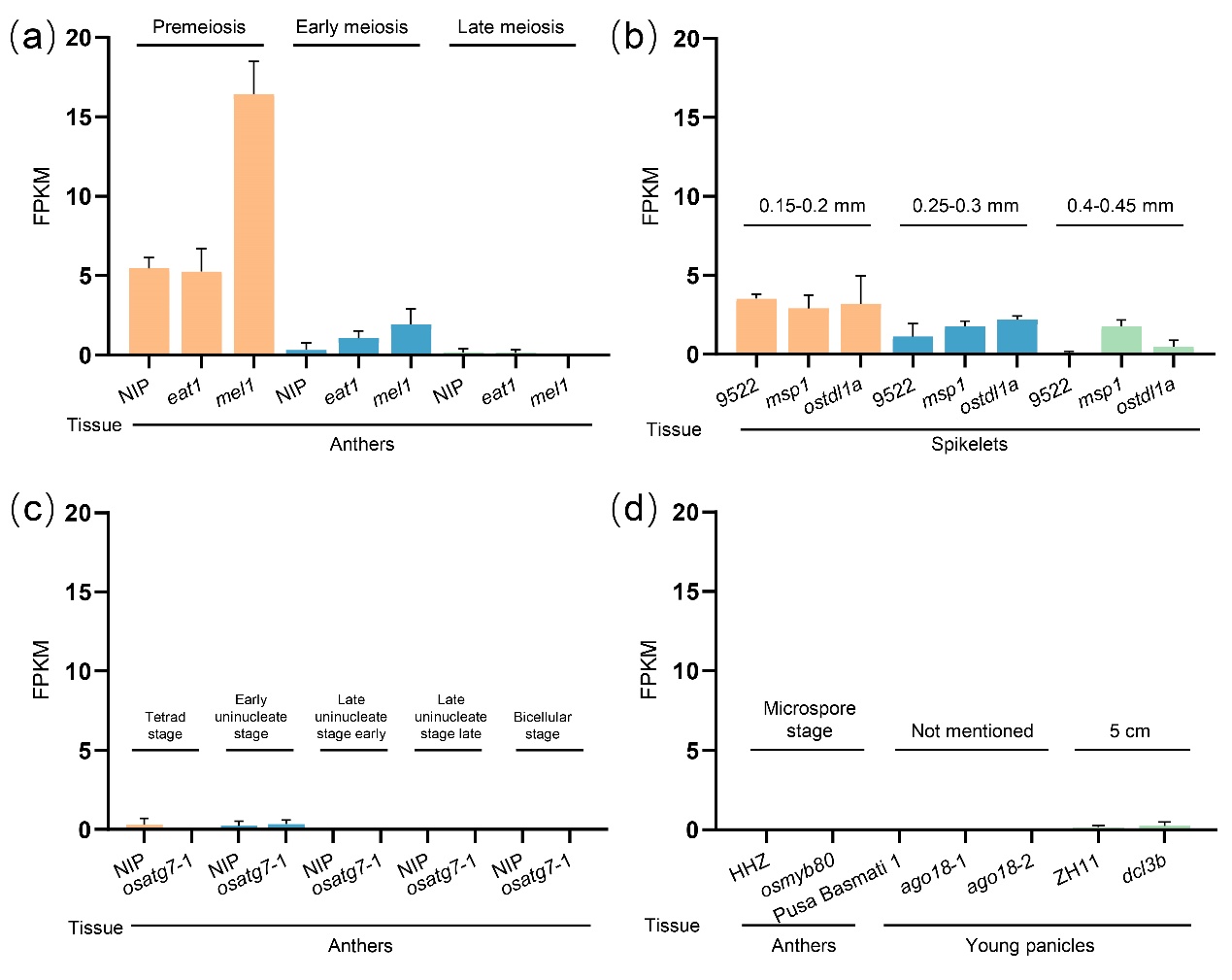


**Figure S8.** The expression level of *sdgms* in rice male sterility mutants in the plant public RNA-seq Database (<http://ipf.sustech.edu.cn/pub/plantrna/?lngdjecbaiecjecj>). (a) The expression level of *sdgms* in *mel*-1, *eat1*, and NIP anthers. Relative to NIP, *sdgms* was upregulated in *mel1* anthers at the premeiosis and early meiosis stages. (b) The expression level of *sdgms* in *msp1*, *ostdl1*, and 9522 spikelets. (c) The expression level of *sdgms* in *osatg7-1* mutant and NIP anthers. (d) The expression level of *sdgms* in *osmyb80*, *ago18* mutant, and *dcl3b* mutants compared with their wild-type parents.

**Table S1.** Spikelet fertility and segregation ratio in the F_1_ plants from crosses between three *SDGMS* NILs and their fertile male parents.

| **Cross** | **Year** | **Number of plants** | |  | **Spikelet fertility (%)** | | ***P* value** | ***χ*^2^(1:1)** |
| --- | --- | --- | --- | --- | --- | --- | --- | --- |
|  |  | Sterile | Fertile |  | Sterile | Fertile |  |  |
| 938(*SDGMS*) × 938 | 2015-HN | 204 | 189 |  | 0 | 89.06±1.15 | 1.73E-24 | 0.57 |
|  | 2015-WH | 353 | 341 |  | 0 | 85.47±1.99 | 7.12E-20 | 0.21 |
| NIP(SDGMS) × NIP | 2015-WH | 43 | 48 |  | 0 | 70.04±2.67 | 4.32E-16 | 0.27 |
|  | 2016-HN | 21 | 27 |  | 0 | 67.91±2.83 | 2.03E-15 | 0.75 |
| ZS97(SDGMS) × ZS97 | 2019-HN | 73 | 66 |  | 0 | 83.12±1.92 | 5.98E-20 | 0.35 |
|  | 2019-WH | 92 | 99 |  | 0 | 79.99±1.66 | 9.00E-21 | 0.26 |

HN indicates the plants planted in Hainan Province, and WH indicates the plants planted in Wuhan. The segregation ratios are tested by the chi-square test. The data presented are the mean ± S.E.M. *P* values were obtained from *t* tests between the sterile and fertile plants.

**Table S2.** A total of 330 rice accessions worldwide were investigated and none of them carries the 1978-bp insertion.

| **Accession ID** | **Accession Name** | **Subpopulation** | **Location** |
| --- | --- | --- | --- |
| W001 | Mayang Khang | *Indica* I | Indonesia |
| W002 | E B Gopher | Tropical *Japonica* | United States |
| W003 | C 5560 | Tropical *Japonica* | Thailand |
| W004 | Quinimpol | Tropical *Japonica* | Philippines |
| W005 | TAICHU MOCHI 59 | Tropical *Japonica* | China |
| W006 | WC 2811 | Tropical *Japonica* | Micronesia |
| W007 | Criollo Chivacoa 2 | Tropical *Japonica* | Venezuela |
| W008 | Secano do Brazil | Tropical *Japonica* | El Salvador |
| W009 | BERLIN | *Indica* III | Costa Rica |
| W010 | Sel. No. 388 | *Japonica* Intermediate | Uruguay |
| W011 | SHIMIZU MOCHI | Temperate *Japonica* | Japan |
| W012 | KRASNODARSKIJ 424 | Temperate *Japonica* | Russian Federation |
| W013 | Pergonil 15 | Temperate *Japonica* | Portugal |
| W014 | Red Khosha Cerma | *VI*/*Aromatic* | Afghanistan |
| W015 | Safut Khosha | *Aus* | Afghanistan |
| W016 | NORIN 11 | Temperate *Japonica* | Japan |
| W017 | R 75 | Tropical *Japonica* | Senegal |
| W018 | UZ ROSZ M38 | Temperate *Japonica* | Uzbekistan |
| W019 | H57-3-1 | *Japonica* Intermediate | Argentina |
| W020 | IARI 6626 | *Aus* | India |
| W021 | Khao Luang | *Japonica* Intermediate | Laos |
| W022 | C 8429 | Tropical *Japonica* | Papua New Guinea |
| W023 | Warrangal Culture 1252 | *Indica* III | India |
| W024 | Padi Pohon Batu | Tropical *Japonica* | Malaysia |
| W025 | NC 1/536 | *Aus* | Pakistan |
| W026 | Won Son Zo No. 11 | *Indica* I | Korea |
| W027 | Chacareiro Uruguay | Temperate *Japonica* | Uruguay |
| W028 | Doble Carolina | *Aus* | Uruguay |
| W029 | Ai Chueh Ta Pai Ku | *Indica* Intermediate | China |
| W030 | Thang 10 | *Indica* Intermediate | *VI*etnam |
| W031 | Sipirasikkam | *Indica* I | Indonesia |
| W032 | TJ | *Indica* I | Guyana |
| W033 | PD 46 | *Indica* I | Sri Lanka |
| W034 | PATNAI 6 | *Aus* | Myanmar |
| W035 | K8C-263-3 | *Indica* Intermediate | Suriname |
| W036 | Chun 118-33 | *Indica* I | China |
| W037 | Manga Kely 694 | *Indica* Intermediate | Madagascar |
| W038 | BLUE STICK | Temperate *Japonica* | Fiji |
| W039 | Nam Dawk Mai | *Indica* I | Thailand |
| W040 | INIAP 7 | *Indica* II | Ecuador |
| W041 | Onu B | Tropical *Japonica* | Zaire |
| W042 | Red | *Indica* Intermediate | Pakistan |
| W043 | Dichroa Alef Uslkij | *Indica* Intermediate | Kazakhstan |
| W044 | BKN 6987-68-14 | *Indica* II | Thailand |
| W045 | KUBANETS 508 | Temperate *Japonica* | Russian |
| W046 | IR 9660-48-1-1-2 | *Indica* II | Philippines |
| W047 | Jumli dhan | *Japonica* Intermediate | Nepal |
| W048 | N-2703 | *Aus* | Nepal |
| W049 | PHUDUGEY | *Aus* | Bhutan |
| W050 | Ak Tokhum | *VI*/*Aromatic* | Azerbaijan |
| W051 | RP2151-173-1-8 | *Indica* II | India |
| W052 | HB-6-2 | Temperate *Japonica* | Hungary |
| W053 | GPNO 1106 | Tropical *Japonica* | Guatemala |
| W054 | Toga | *Indica* Intermediate | India |
| W055 | Ragasu | Intermediate | China |
| W056 | Tamanishiki | Temperate *Japonica* | Japan |
| W057 | Grassy | Tropical *Japonica* | Haiti |
| W058 | Kao Chio Lin Chou | *Indica* I | China |
| W059 | Niwahutaw Mochi | Temperate *Japonica* | Japan |
| W060 | Somewake | Temperate *Japonica* | Japan |
| W061 | Ardito | *Japonica* Intermediate | Italy |
| W062 | NANTON NO. 131 | Tropical *Japonica* | China |
| W063 | Vary Tarva Osla | *Japonica* Intermediate | Portugal |
| W064 | CSORNUJ | Temperate *Japonica* | Hungary |
| W065 | R 67 | Tropical *Japonica* | Senegal |
| W066 | IR 238 | *Indica* Intermediate | Philippines |
| W067 | Mitak | Tropical *Japonica* | Indonesia |
| W068 | Gazan | Temperate *Japonica* | Afghanistan |
| W069 | 99216 | *Aus* | India |
| W070 | Shui Ya Jien | *Indica* Intermediate | China |
| W071 | AKP 4 | *Indica* Intermediate | India |
| W072 | SORNAVARI | *Aus* | Mali |
| W073 | IR 2061-214-2-3 | *Indica* II | Philippines |
| W074 | TAINUNG 45 | *Indica* Intermediate | China |
| W075 | Sapundali Local | *Indica* Intermediate | India |
| W076 | Tauli | *Aus* | Nepal |
| W077 | 79 | *VI*/*Aromatic* | Guyana |
| W078 | B805D-MR-16-8-3 | *Indica* Intermediate | Indonesia |
| W079 | A 152 | Intermediate | Bangladesh |
| W080 | UZ ROS 59 | *Indica* I | Uzbekistan |
| W081 | Gasym Hany | *VI*/*Aromatic* | Azerbaijan |
| W082 | Celiaj | Temperate *Japonica* | Azerbaijan |
| W083 | CNTLR80076-44-1-1-1 | *Indica* Intermediate | Thailand |
| W084 | IR 58614-B-B-8-2 | *Indica* Intermediate | Philippines |
| W085 | KECHENGNUO NO. 4 | *Indica* Intermediate | China |
| W086 | 4484 | *Indica* II | China |
| W087 | YOU-I B | *Indica* I | China |
| W088 | CHUNJIANGZAO NO. 1 | Temperate *Japonica* | China |
| W089 | Egyptian Wild Type | *Indica* III | Turkey |
| W090 | C.B. II | *Aus* | Japan |
| W091 | Gallawa | *Aus* | Sri Lanka |
| W092 | Karayal | *Aus* | Sri Lanka |
| W093 | Srav Prapay | *Indica* III | Cambodia |
| W094 | Nang Bang Bentre | *Aus* | *VI*etnam |
| W095 | DJ 24 | *Aus* | Bangladesh |
| W096 | DJ 102 | *Aus* | Bangladesh |
| W097 | Santhi 990 | Intermediate | Pakistan |
| W098 | UZ ROS 7-13 | *Aus* | Uzbekistan |
| W099 | SL 22-620 | *Aus* | Sierra Leone |
| W100 | Spin Mere | *Aus* | Afghanistan |
| W101 | AMANE | *Indica* Intermediate | Sri Lanka |
| W102 | Padi Tarab Arab | Tropical *Japonica* | Malaysia |
| W103 | P 35 | *Aus* | India |
| W104 | CAROLINO 164 | *Aus* | Chad |
| W105 | HKG 98 | *Aus* | Mali |
| W106 | Daudzai Field Mix | *Aus* | Pakistan |
| W107 | JP 5 | *Indica* Intermediate | Pakistan |
| W108 | Hi Muke | *Aus* | Kazakhstan |
| W109 | WIR 911 | Temperate *Japonica* | Russian Federation |
| W110 | Lua Chua Chan | Tropical *Japonica* | *VI*etnam |
| W111 | Sereno | *Indica* III | Jamaica |
| W112 | ARC 10633 | *Indica* III | India |
| W113 | Simpor | Tropical *Japonica* | Brunei |
| W114 | Heo Trang | *Indica* III | *VI*etnam |
| W115 | THAVALU | *Aus* | Sri Lanka |
| W116 | WC 10253 | Tropical *Japonica* | Philippines |
| W117 | KRASNODARSKIJ 3352 | Temperate *Japonica* | Russian Federation |
| W118 | EMBRAPA 1200 | Tropical *Japonica* | Brazil |
| W119 | WAB462-10-3-1 | Tropical *Japonica* | Cote D'Ivoire |
| W120 | Bombilla | Temperate *Japonica* | Spain |
| W121 | TCHAMPA | *Aus* | Iran |
| W122 | BHIM DHAN | *Japonica* Intermediate | Nepal |
| W123 | WC 3532 | Tropical *Japonica* | Peru |
| W124 | Kin Shan Zim | *Indica* I | China |
| W125 | Yong Chal Byo | Intermediate | South Korea |
| W126 | Pan Ju | *Indica* I | China |
| W127 | Buphopa | *Japonica* Intermediate | Myanmar |
| W128 | TAINO 38 | Intermediate | China |
| W129 | 2 | *VI*/*Aromatic* | Afghanistan |
| W130 | LUSITANO | Temperate *Japonica* | Portugal |
| W131 | IARI 6621 | *Aus* | India |
| W132 | Hsin Hsing Pai Ku | *Indica* I | China |
| W133 | TD 70 | *Indica* Intermediate | Thailand |
| W134 | Dara | *Aus* | Indonesia |
| W135 | Shimla Early | *Indica* Intermediate | Iraq |
| W136 | LA PLATA GENA F.A. | *VI*/*Aromatic* | Argentina |
| W137 | CM1; HAIPONG | *Indica* Intermediate | *VI*etnam |
| W138 | 4595 | *Indica* I | China |
| W139 | A 5 | Temperate *Japonica* | Japan |
| W140 | Ittikulama | *Aus* | Sri Lanka |
| W141 | DNJ 179 | *Aus* | Bangladesh |
| W142 | DNJ 121 | *Aus* | Bangladesh |
| W143 | Jyanak | *Japonica* Intermediate | Bhutan |
| W144 | Ao Chiu 2 Hao | *Indica* I | China |
| W145 | GUYANE 1 | *Indica* Intermediate | Chad |
| W146 | 10340 | *Indica* I | Italy |
| W147 | BR11 | *Indica* Intermediate | Bangladesh |
| W148 | BR24 | *Indica* Intermediate | India |
| W149 | 93072 | *Indica* Intermediate | China |
| W150 | 108S | *Indica* II | China |
| W151 | C418 | *Indica* II | China |
| W152 | CDR22 | *Indica* II | China |
| W153 | Chenghui448 | *Indica* II | China |
| W154 | Fengaizhan | *Indica* II | China |
| W155 | Gang46B | *Indica* Intermediate | China |
| W156 | Gumei2hao | *Indica* I | China |
| W157 | Huajingxian74 | *Indica* Intermediate | China |
| W158 | M3122 | Intermediate | China |
| W159 | R644 | *Indica* II | China |
| W160 | Shennong265 | Temperate *Japonica* | China |
| W161 | Y134 | *Indica* II | China |
| W162 | Yuanjing7 | Temperate *Japonica* | China |
| W163 | Yuexiangzhan | *Indica* Intermediate | China |
| W164 | Zaoxian14 | *Indica* Intermediate | China |
| W165 | Zhong413 | *Indica* Intermediate | China |
| W166 | Zhongyouzao81 | *Indica* Intermediate | China |
| W167 | Zihui100 | *Indica* Intermediate | China |
| W168 | Bg90-2 | *Indica* II | Brazil |
| W169 | IR72 | *Indica* II | Philippines |
| W170 | PR106 | *Indica* II | Pakistan |
| W171 | TKM9 | *Indica* Intermediate | Philippines |
| W172 | Amo13 (Sana) | *Indica* II | Laos |
| W173 | Khazar | Tropical *Japonica* | Iran |
| W174 | Gayabyeo | *Indica* II | Korea |
| W175 | Iksan438 | Intermediate | Korea |
| W176 | Milyang23 | *Indica* II | Korea |
| W177 | MR185 | *Indica* II | Malaysia |
| W178 | Manawthukha | *Indica* III | Philippines |
| W179 | Shwe Thwe Yin Hyv | *Indica* II | Myanmar |
| W180 | Bg300 | *Indica* Intermediate | Sri Lanka |
| W181 | Bg94-1 | *Indica* Intermediate | Sri Lanka |
| W182 | CR203 | *Indica* II | *VI*etnam |
| W183 | OM997 | *Indica* II | *VI*etnam |
| W184 | PSB RC 28 | *Indica* II | Philippines |
| W185 | PSB RC 66 | *Indica* II | Philippines |
| W186 | TEQING | *Indica* Intermediate | Philippines |
| W187 | IR68552-55-3-2 | *Indica* Intermediate | Philippines |
| W188 | IR66897B | *Indica* II | Philippines |
| W189 | IR58025B | *Indica* II | Philippines |
| W190 | Dhan4 | *Indica* II | India |
| W191 | Pusa (Basmatil) | Intermediate | China |
| W192 | ASD16 | Intermediate | India |
| W193 | Dianjing | Temperate *Japonica* | China |
| W194 | F6 | *Indica* II | Japan |
| W195 | 452 | *Indica* II | Unknown |
| W196 | NAN-29-2 | Intermediate | China |
| W197 | Ai-Zi-DAO | *Indica* II | China |
| W198 | Babaomi | *Indica* Intermediate | China |
| W199 | Diantun502 | *Indica* Intermediate | China |
| W200 | Heiheaihui | *Indica* Intermediate | China |
| W201 | Jiangxisimiao | *Indica* Intermediate | China |
| W202 | Laohudao | *Indica* Intermediate | China |
| W203 | Linjintangdao | *Indica* Intermediate | China |
| W204 | Mengguandamagu | *Indica* I | China |
| W205 | Pengshantieganzhan | *Indica* I | China |
| W206 | Wumanggaonuo | *Indica* Intermediate | China |
| W207 | Yunguang8hao | Temperate *Japonica* | China |
| W208 | Zhongchao123 | Temperate *Japonica* | China |
| W209 | Gizal 159 | Temperate *Japonica* | EGYPT |
| W210 | Khao Daeng | *Indica* I | Philippines |
| W211 | Basmati370 | *Indica* II | Philippines |
| W212 | Bhavani | *Indica* Intermediate | India |
| W213 | IR50 | *Indica* Intermediate | India |
| W214 | Jhona349 | *Aus* | Pakistan |
| W215 | Karnal Local | *Aus* | India |
| W216 | Type3 | *Indica* II | India |
| W217 | IRAT352 | *Indica* Intermediate | Indonesia |
| W218 | TB154E-TB-2 | *Indica* Intermediate | Indonesia |
| W219 | Domsiah | *VI*/*Aromatic* | Netherlands |
| W220 | Tarom molai 1 | *VI*/*Aromatic* | Iran |
| W221 | MR77 (seberang) | *Indica* Intermediate | Philippines |
| W222 | ir6 | *Indica* II | Pakistan |
| W223 | At354 | *Indica* II | Sri Lanka |
| W224 | BG304 | *Indica* II | Sri Lanka |
| W225 | Lemont | Tropical *Japonica* | Philippines |
| W226 | M401 | Temperate *Japonica* | Philippines |
| W227 | OM1706 | *Indica* Intermediate | *VI*etnam |
| W228 | OM1723 | *Indica* II | *VI*etnam |
| W229 | X21 | *Indica* II | *VI*etnam |
| W230 | X22 | *Indica* II | *VI*etnam |
| W231 | X23 | *Indica* II | *VI*etnam |
| W232 | C71 | Temperate *Japonica* | *VI*etnam |
| W233 | C70 | *Indica* Intermediate | *VI*etnam |
| W234 | Q5 | *Indica* Intermediate | *VI*etnam |
| W235 | Suiyangnian | *Indica* I | China |
| W236 | Pokhreli | *Indica* II | Philippines |
| W237 | Govnd | *Indica* II | Philippines |
| W238 | UPR191-66 | *Indica* II | India |
| W239 | ASD18 | *Indica* Intermediate | India |
| W240 | TGMS29 | *Indica* Intermediate | India |
| W241 | Phalguna | *Indica* II | India |
| W242 | Ajaya | *Indica* Intermediate | India |
| W243 | Dodda | *Indica* II | India |
| W244 | Palung 2 | *Indica* II | Philippines |
| W245 | TKM6 | *Indica* Intermediate | India |
| W246 | RUSTYLATE/Zhong 413 | *Indica* Intermediate | China |
| W247 | Yenfangghu | *Indica* I | China |
| W248 | Heimichut | *Indica* I | China |
| W249 | Teksichut | Temperate *Japonica* | Philippines |
| W250 | Eiko | Temperate *Japonica* | Japan |
| W251 | RUBIO | Tropical *Japonica* | Philippines |
| W252 | SADRI RICE 1 | *VI*/*Aromatic* | Iran |
| W253 | Sereendan Kuning | Tropical *Japonica* | Malaysia |
| W254 | SAI-BUI-BAO | Tropical *Japonica* | *VI*etnam |
| W255 | ZIRI | *VI*/*Aromatic* | India |
| W256 | Dacca6 | *VI*/*Aromatic* | United States |
| W257 | Latisai1 | Tropical *Japonica* | Bangladesh |
| W258 | Amareles | *VI*/*Aromatic* | Portugal |
| W259 | Up15 | *Indica* II | Japan |
| W260 | Uz-Rosz 275 | Tropical *Japonica* | Philippines |
| W261 | Ginga | Intermediate | Japan |
| W262 | Bintapan | *Indica* I | Philippines |
| W263 | SADAJIRA-19-303 | *Indica* Intermediate | Bangladesh |
| W264 | CHOROFA | *Indica* Intermediate | Philippines |
| W265 | La110 | *Indica* Intermediate | United States |
| W266 | Yunjiang35 | *Indica* III | China |
| W267 | Yunhui72 | *Indica* Intermediate | China |
| W268 | Wudadaozhong | *Indica* Intermediate | South Korea |
| W269 | IR65600-27-1-2-2 | *Indica* Intermediate | Philippines |
| W270 | Yetuozai | *Indica* Intermediate | China |
| W271 | P59279 | *Indica* Intermediate | Pakistan |
| W272 | Tsao wan ching | *Indica* Intermediate | China |
| W273 | Anambae ndanggalasi | Tropical *Japonica* | Indonesia |
| W274 | Er chiu ching | *Indica* I | China |
| W275 | Heen goda wee | Tropical *Japonica* | Sri Lanka |
| W276 | Pathma wee | *Indica* III | Sri Lanka |
| W277 | Lakkhi kajal | *Aus* | Bangladesh |
| W278 | Moisdol | *Aus* | Bangladesh |
| W279 | Jabor sail | *Aus* | Bangladesh |
| W280 | Sada solay | *Aus* | Pakistan |
| W281 | Sholay | Temperate *Japonica* | Pakistan |
| W282 | Ma ba you zhan | *Indica* Intermediate | China |
| W283 | Daegujo | Temperate *Japonica* | Republic of Korea |
| W284 | Paikasa | Tropical *Japonica* | Malaysia |
| W285 | Uwi | Tropical *Japonica* | Indonesia |
| W286 | Garia | *Aus* | Bangladesh |
| W287 | AUS 449 | *Aus* | Bangladesh |
| W288 | Dourado Precoce | Tropical *Japonica* | Brazil |
| W289 | IR 661-1-140-3-117 | *Indica* II | Philippines |
| W290 | Aichi Asahi | Temperate *Japonica* | Japan |
| W291 | IR 2071-625-1-252 | *Indica* II | Philippines |
| W292 | Khoia boro | *Aus* | Bangladesh |
| W293 | IR 8 | *Indica* II | Philippines |
| W294 | LABELLE | Intermediate | United States |
| W295 | LEBONNET | *Indica* II | United States |
| W296 | *AUS* 371 | *Aus* | Bangladesh |
| W297 | *AUS* 373 | *Aus* | Bangladesh |
| W298 | IR 36 | *Indica* II | Philippines |
| W299 | JAYA | *Indica* Intermediate | India |
| W300 | NEW BONNET | Tropical *Japonica* | United States |
| W301 | IR 64 | *Indica* II | Philippines |
| W302 | KUNTLAN | Tropical *Japonica* | Indonesia |
| W303 | British Honduras Creole | Tropical *Japonica* | Belize |
| W304 | CPSLO 17 | Intermediate | United States |
| W305 | AKITAKOMACHI | Temperate *Japonica* | Japan |
| W306 | BASMATI 385 | *VI*/*Aromatic* | Pakistan |
| W307 | AGNO (PSBRC28) | *Indica* II | Philippines |
| W308 | PSBRC82 | *Indica* II | Philippines |
| W309 | NSICRC122 | *Indica* II | Philippines |
| W310 | BRRI DHAN 29 | *Indica* Intermediate | Bangladesh |
| W311 | PR 116 | *Indica* II | India |
| W312 | CIGEULIS | *Indica* II | Indonesia |
| W313 | OM 2517 | *Indica* II | *VI*etnam |
| W314 | BRRI DHAN 28 | *Indica* II | Bangladesh |
| W315 | CIBOGO | *Indica* II | Indonesia |
| W316 | NEDA | *Indica* II | Islamic Republic of Iran |
| W317 | CIHERANG | *Indica* II | Indonesia |
| W318 | Qiuqianbai | *Indica* I | China |
| W319 | Guangkexiangnuo | Temperate *Japonica* | China |
| W320 | Wenxiangnuo | *Indica* Intermediate | China |
| W321 | Laozaogu | *Indica* III | China |
| W322 | Lengshuinuo | *Indica* Intermediate | China |
| W323 | Feienuo2 | *Japonica* Intermediate | China |
| W324 | Zimangfeie | *Japonica* Intermediate | China |
| W325 | Guantuibaihe | Temperate *Japonica* | China |
| W326 | Haolvguangnian | *Japonica* Intermediate | China |
| W327 | JinghuB | Temperate *Japonica* | China |
| W328 | Sibeitichao6 | Intermediate | China |
| W329 | GPNO 5055 | Tropical *Japonica* | United States |
| W330 | ASWINA 330 | *Aus* | Bangladesh |

**Table S3.** Spikelet fertility of the transgenic lines.

| **Transgenic lines** | **Line or cross** | **Generation** | **Genotype** | **No. of plants** | **Spikelet**  **fertility (%)** | ***P* values** |
| --- | --- | --- | --- | --- | --- | --- |
| *SDGM*S-N | *SDGMS*-N(T_0_) | T_0_ | (+) | 17 | 13.48±2.78 | 1.52E-11 |
|  |  |  | (-) | 8 | 65.34±1.67 | *** |
|  | *SDGMS*-N(T_1_-1) | T_1_ | (+) | 23 | 9.73±1.88 | 2.68E-16 |
|  |  |  | (-) | 7 | 70.64±2.42 | *** |
|  | *SDGMS*-N(T_1_-2) | T_1_ | (+) | 20 | 9.61±1.70 | 7.02E-16 |
|  |  |  | (-) | 10 | 64.88±3.56 | *** |
|  | *SDGMS*-N(BC_1_-1) | BC1(T_0_×NIP) | (+) | 16 | 2.90±0.95 | 3.01E-16 |
|  |  |  | (-) | 7 | 70.7±3.95 | *** |
|  | *SDGMS*-N(BC_1_-2) | BC1(T_0_×NIP) | (+) | 18 | 5.72±1.74 | 1.92E-13 |
|  |  |  | (-) | 5 | 62.1±2.37 | *** |
| *SDGMS*-OE | *SDGMS*-OE(T_0_) | T_0_ | (+) | 26 | 0 | - |
|  |  |  | (-) | 0 | - | - |
|  | *SDGMS*-OE(BC_1_-1) | BC1(T_0_×NIP) | (+) | 16 | 0 | 9.37E-26 |
|  |  |  | (-) | 15 | 67.93±1.98 | *** |
|  | *SDGMS*-OE(BC_1_-2) | BC1(T_0_×NIP) | (+) | 18 | 0 | 9.94E-28 |
|  |  |  | (-) | 16 | 67.43±2.00 | *** |
|  | *SDGMS*-OE(BC_1_-3) | BC1(T_0_×NIP) | (+) | 25 | 0 | 2.87E-29 |
|  |  |  | (-) | 11 | 69.00±2.88 | *** |
| *SDGMS^ko^* | *SDGMS^ko^* -1 | T_0_ | (-2_+28)  30-bp deletion | - | 67.36 | - |
|  | *SDGMS^ko^* -2 | T_0_ | (-13_+15)  28-bp deletion | - | 71.70 | - |
|  | *SDGMS^ko^* -3 | T_0_ | (-62_+4)  66-bp deletion | - | 62.27 | - |
|  | *SDGMS^ko^* -4 | T_0_ | (-1)  1-bp insertion | - | 0 | - |
|  | *SDGMS^ko^* -5 | T_0_ | (-3)  2-bp insertion | - | 0 | - |
|  | *SDGMS^ko^* -6 | T_0_ | (-9_-2)  8-bp deletion | - | 0 | - |
|  | *SDGMS^ko^*-1(T_1_) | T_1_ | *SDGMS^ko^* | 19 | 84.50±0.91 | 0.2571 |
|  | from fertile T_0_ |  | *sdgms* | 10 | 83.39±1.57 | NS |
|  | *SDGMS^ko^*-2(T_1_) | T_1_ | *SDGMS^ko^* | 21 | 82.56±0.94 | 0.1434 |
|  | from fertile T_0_ |  | *sdgms* | 9 | 84.23±0.79 | NS |
|  | *SDGMS^ko^*-3(T_1_) | T_1_ | *SDGMS^ko^* | 22 | 84.58±0.91 | 0.1755 |
|  | from fertile T_0_ |  | *sdgms* | 8 | 82.91±1.49 | NS |
|  | *SDGMS^ko^*-6(BC_1_) | BC_1_(T_0_×ZS97) | *SDGMS^ko^* | 17 | 0.57±0.21 | 3.45E-36 |
|  | from sterile T_0_ |  | *sdgms* | 15 | 82.77±1.13 | *** |
| *sdgms*-OE | *sdgms*-OE(T_1_-1) | T_1_ | (+) | 18 | 61.06±2.12 | 0.0921 |
|  |  |  | (-) | 6 | 66.75±3.32 | NS |
|  | *sdgms*-OE(T_1_-2) | T_1_ | (+) | 18 | 59.33±1.70 | 0.2800 |
|  |  |  | (-) | 4 | 61.79±4.45 | NS |
|  | *sdgms*-OE(T_1_-3) | T_1_ | (+) | 14 | 55.61±2.30 | 0.2866 |
|  |  |  | (-) | 4 | 58.41±4.30 | NS |

All data presented are the mean ± S.E.M. The numbers within the parentheses indicate the position of the mutations from the start codon ATG. (+) and (-) indicate transgenic positive and negative plants, respectively. *P* values were obtained from *t* tests between transgenic positive and negative plants.

**Table S4.** Differentially expressed genes (DEGs) detected in 938(*SDGMS*) and 938(*sdgms*).

(data are presented as an excel file)

DEGs were obtained using the DESeq2. BaseMean indicates the relative expression level of genes; Log2FoldChange > 1 indicates the genes that upregulated in 938(*SDGMS*) and Log2FoldChange < -1 indicates the genes that downregulated in 938(*SDGMS*); Adj *P* value indicates adjust *P* value.

**Table S5.** The enriched GO terms in EM and LM.

| **GO_ID** | **Type** | **Term** | **Query**  **item** | **Background item** | **FDR** |
| --- | --- | --- | --- | --- | --- |
| **The Enriched GO terms of upregulated genes in EM** | | | | | |
| GO:0009765 | P | photosynthesis, light harvesting | 5 | 16 | 2.70E-05 |
| GO:0010467 | P | gene expression | 30 | 2424 | 0.00045 |
| GO:0019684 | P | photosynthesis, light reaction | 5 | 47 | 0.001 |
| GO:0009889 | P | regulation of biosynthetic process | 21 | 1564 | 0.0022 |
| GO:0010556 | P | regulation of macromolecule biosynthetic process | 21 | 1564 | 0.0022 |
| GO:0031326 | P | regulation of cellular biosynthetic process | 21 | 1564 | 0.0022 |
| GO:0010468 | P | regulation of gene expression | 21 | 1577 | 0.0022 |
| GO:0031323 | P | regulation of cellular metabolic process | 21 | 1593 | 0.0023 |
| GO:0080090 | P | regulation of primary metabolic process | 21 | 1626 | 0.0023 |
| GO:0060255 | P | regulation of macromolecule metabolic process | 21 | 1640 | 0.0023 |
| GO:0045449 | P | regulation of transcription | 20 | 1513 | 0.0023 |
| GO:0019219 | P | regulation of nucleobase, nucleoside, nucleotide and nucleic acid metabolic process | 20 | 1520 | 0.0023 |
| GO:0051171 | P | regulation of nitrogen compound metabolic process | 20 | 1520 | 0.0023 |
| GO:0019222 | P | regulation of metabolic process | 21 | 1664 | 0.0024 |
| GO:0009607 | P | response to biotic stimulus | 3 | 16 | 0.0038 |
| GO:0009308 | P | amine metabolic process | 9 | 380 | 0.0044 |
| GO:0006350 | P | transcription | 20 | 1629 | 0.0044 |
| GO:0006520 | P | cellular amino acid metabolic process | 8 | 303 | 0.0045 |
| GO:0050794 | P | regulation of cellular process | 22 | 1955 | 0.0061 |
| GO:0044106 | P | cellular amine metabolic process | 8 | 333 | 0.0076 |
| GO:0015979 | P | photosynthesis | 5 | 115 | 0.0082 |
| GO:0043038 | P | amino acid activation | 4 | 66 | 0.0086 |
| GO:0050789 | P | regulation of biological process | 22 | 2027 | 0.0086 |
| GO:0043039 | P | tRNA aminoacylation | 4 | 66 | 0.0086 |
| GO:0006418 | P | tRNA aminoacylation for protein translation | 4 | 66 | 0.0086 |
| GO:0065007 | P | biological regulation | 22 | 2125 | 0.014 |
| GO:0006519 | P | cellular amino acid and derivative metabolic process | 8 | 391 | 0.033 |
| GO:0006412 | P | translation | 10 | 595 | 0.015 |
| GO:0016070 | P | RNA metabolic process | 14 | 1088 | 0.018 |
| GO:0006548 | P | histidine catabolic process | 2 | 8 | 0.019 |
| GO:0009077 | P | histidine family amino acid catabolic process | 2 | 8 | 0.019 |
| **The Enriched GO terms of downregulated genes in EM** | | | | | |
| - | | | | | |
| **The Enriched GO terms of upregulated genes in LM** | | | | | |
| GO:0006468 | P | protein amino acid phosphorylation | 108 | 1570 | 1.30E-13 |
| GO:0016310 | P | phosphorylation | 109 | 1695 | 3.50E-12 |
| GO:0043687 | P | post-translational protein modification | 113 | 1815 | 5.60E-12 |
| GO:0006796 | P | phosphate metabolic process | 111 | 1795 | 9.20E-12 |
| GO:0006793 | P | phosphorus metabolic process | 111 | 1795 | 9.20E-12 |
| GO:0006464 | P | protein modification process | 114 | 1929 | 5.70E-11 |
| GO:0043412 | P | macromolecule modification | 114 | 1979 | 2.40E-10 |
| GO:0009765 | P | photosynthesis, light harvesting | 12 | 16 | 4.10E-10 |
| GO:0045449 | P | regulation of transcription | 84 | 1513 | 1.10E-06 |
| GO:0019219 | P | regulation of nucleobase, nucleoside, nucleotide and nucleic acid metabolic process | 84 | 1520 | 1.10E-06 |
| GO:0051171 | P | regulation of nitrogen compound metabolic process | 84 | 1520 | 1.10E-06 |
| GO:0009889 | P | regulation of biosynthetic process | 84 | 1564 | 2.90E-06 |
| GO:0010556 | P | regulation of macromolecule biosynthetic process | 84 | 1564 | 2.90E-06 |
| GO:0031326 | P | regulation of cellular biosynthetic process | 84 | 1564 | 2.90E-06 |
| GO:0019684 | P | photosynthesis, light reaction | 12 | 47 | 3.60E-06 |
| GO:0010468 | P | regulation of gene expression | 84 | 1577 | 3.60E-06 |
| GO:0055114 | P | oxidation reduction | 75 | 1351 | 4.00E-06 |
| GO:0031323 | P | regulation of cellular metabolic process | 84 | 1593 | 4.90E-06 |
| GO:0080090 | P | regulation of primary metabolic process | 85 | 1626 | 5.50E-06 |
| GO:0060255 | P | regulation of macromolecule metabolic process | 85 | 1640 | 7.40E-06 |
| GO:0006350 | P | transcription | 84 | 1629 | 1.00E-05 |
| GO:0019222 | P | regulation of metabolic process | 85 | 1664 | 1.20E-05 |
| GO:0009607 | P | response to biotic stimulus | 7 | 16 | 7.60E-05 |
| GO:0044267 | P | cellular protein metabolic process | 124 | 2877 | 0.00011 |
| GO:0050794 | P | regulation of cellular process | 90 | 1955 | 0.00029 |
| GO:0050789 | P | regulation of biological process | 91 | 2027 | 0.00062 |
| GO:0065007 | P | biological regulation | 94 | 2125 | 0.00074 |
| GO:0050832 | P | defense response to fungus | 4 | 4 | 0.001 |
| GO:0051707 | P | response to other organism | 4 | 4 | 0.001 |
| GO:0009620 | P | response to fungus | 4 | 4 | 0.001 |
| GO:0009617 | P | response to bacterium | 4 | 4 | 0.001 |
| GO:0042742 | P | defense response to bacterium | 4 | 4 | 0.001 |
| GO:0015979 | P | photosynthesis | 13 | 115 | 0.0015 |
| GO:0050896 | P | response to stimulus | 52 | 1026 | 0.0021 |
| GO:0006952 | P | defense response | 29 | 452 | 0.0022 |
| GO:0000272 | P | polysaccharide catabolic process | 7 | 37 | 0.0046 |
| GO:0006030 | P | chitin metabolic process | 6 | 26 | 0.0046 |
| GO:0006032 | P | chitin catabolic process | 6 | 26 | 0.0048 |
| GO:0006026 | P | aminoglycan catabolic process | 6 | 26 | 0.0048 |
| GO:0051234 | P | establishment of localization | 72 | 1638 | 0.0057 |
| GO:0006810 | P | transport | 72 | 1638 | 0.0057 |
| GO:0051179 | P | localization | 72 | 1651 | 0.0069 |
| GO:0006022 | P | aminoglycan metabolic process | 6 | 29 | 0.0073 |
| GO:0019953 | P | sexual reproduction | 5 | 19 | 0.0087 |
| GO:0006915 | P | apoptosis | 30 | 532 | 0.01 |
| GO:0012501 | P | programmed cell death | 30 | 532 | 0.01 |
| GO:0006950 | P | response to stress | 43 | 873 | 0.011 |
| GO:0000003 | P | reproduction | 12 | 133 | 0.013 |
| GO:0008219 | P | cell death | 30 | 544 | 0.013 |
| GO:0016265 | P | death | 30 | 544 | 0.013 |
| GO:0030001 | P | metal ion transport | 16 | 214 | 0.013 |
| GO:0055085 | P | transmembrane transport | 36 | 701 | 0.013 |
| GO:0051704 | P | multi-organism process | 11 | 120 | 0.018 |
| GO:0004672 | F | protein kinase activity | 108 | 1575 | 1.20E-13 |
| GO:0004674 | F | protein serine/threonine kinase activity | 102 | 1478 | 3.20E-13 |
| GO:0016773 | F | phosphotransferase activity, alcohol group as acceptor | 110 | 1726 | 2.30E-12 |
| GO:0032559 | F | adenyl ribonucleotide binding | 162 | 3087 | 2.90E-12 |
| GO:0005524 | F | ATP binding | 162 | 3085 | 2.90E-12 |
| GO:0016301 | F | kinase activity | 110 | 1750 | 2.90E-12 |
| GO:0001883 | F | purine nucleoside binding | 166 | 3247 | 8.40E-12 |
| GO:0030554 | F | adenyl nucleotide binding | 166 | 3247 | 8.40E-12 |
| GO:0001882 | F | nucleoside binding | 166 | 3257 | 9.60E-12 |
| GO:0032555 | F | purine ribonucleotide binding | 164 | 3292 | 7.30E-11 |
| GO:0032553 | F | ribonucleotide binding | 164 | 3292 | 7.30E-11 |
| GO:0017076 | F | purine nucleotide binding | 168 | 3456 | 2.30E-10 |
| GO:0000166 | F | nucleotide binding | 171 | 3874 | 1.90E-07 |
| GO:0005506 | F | iron ion binding | 51 | 713 | 3.80E-07 |
| GO:0020037 | F | heme binding | 45 | 600 | 7.50E-07 |
| GO:0046906 | F | tetrapyrrole binding | 45 | 608 | 1.00E-06 |
| GO:0004497 | F | monooxygenase activity | 37 | 458 | 2.20E-06 |
| GO:0016491 | F | oxidoreductase activity | 87 | 1672 | 3.70E-05 |
| GO:0043565 | F | sequence-specific DNA binding | 35 | 453 | 1.20E-05 |
| GO:0009055 | F | electron carrier activity | 39 | 599 | 0.00013 |
| GO:0003700 | F | transcription factor activity | 38 | 651 | 0.0016 |
| GO:0004568 | F | chitinase activity | 6 | 26 | 0.0065 |
| GO:0004553 | F | hydrolase activity, hydrolyzing O-glycosyl compounds | 26 | 424 | 0.011 |
| GO:0030528 | F | transcription regulator activity | 48 | 987 | 0.011 |
| GO:0016020 | C | membrane | 90 | 2115 | 0.013 |
| **The Enriched GO terms of downregulated genes in LM** | | | | | |
| GO:0006629 | P | lipid metabolic process | 54 | 528 | 6.50E-05 |
| GO:0044257 | P | cellular protein catabolic process | 25 | 153 | 6.50E-05 |
| GO:0006511 | P | ubiquitin-dependent protein catabolic process | 24 | 143 | 6.50E-05 |
| GO:0006633 | P | fatty acid biosynthetic process | 18 | 81 | 6.50E-05 |
| GO:0006631 | P | fatty acid metabolic process | 20 | 97 | 6.50E-05 |
| GO:0051603 | P | proteolysis involved in cellular protein catabolic process | 25 | 153 | 6.50E-05 |
| GO:0043632 | P | modification-dependent macromolecule catabolic process | 24 | 143 | 6.50E-05 |
| GO:0019941 | P | modification-dependent protein catabolic process | 24 | 143 | 6.50E-05 |
| GO:0044281 | P | small molecule metabolic process | 86 | 1034 | 7.70E-05 |
| GO:0051234 | P | establishment of localization | 122 | 1638 | 7.70E-05 |
| GO:0006810 | P | transport | 122 | 1638 | 7.70E-05 |
| GO:0051179 | P | localization | 122 | 1651 | 0.0001 |
| GO:0008610 | P | lipid biosynthetic process | 26 | 180 | 0.00017 |
| GO:0044283 | P | small molecule biosynthetic process | 37 | 320 | 0.00017 |
| GO:0044248 | P | cellular catabolic process | 38 | 337 | 0.0002 |
| GO:0046394 | P | carboxylic acid biosynthetic process | 29 | 222 | 0.0002 |
| GO:0016053 | P | organic acid biosynthetic process | 29 | 222 | 0.0002 |
| GO:0030163 | P | protein catabolic process | 26 | 184 | 0.0002 |
| GO:0009056 | P | catabolic process | 44 | 433 | 0.00031 |
| GO:0055114 | P | oxidation reduction | 101 | 1351 | 0.00036 |
| GO:0009057 | P | macromolecule catabolic process | 35 | 322 | 0.00068 |
| GO:0032787 | P | monocarboxylic acid metabolic process | 20 | 130 | 0.00068 |
| GO:0044255 | P | cellular lipid metabolic process | 28 | 229 | 0.0007 |
| GO:0044265 | P | cellular macromolecule catabolic process | 29 | 254 | 0.0015 |
| GO:0043436 | P | oxoacid metabolic process | 44 | 473 | 0.0017 |
| GO:0019752 | P | carboxylic acid metabolic process | 44 | 473 | 0.0017 |
| GO:0006082 | P | organic acid metabolic process | 44 | 474 | 0.0017 |
| GO:0042180 | P | cellular ketone metabolic process | 44 | 480 | 0.0021 |
| GO:0005975 | P | carbohydrate metabolic process | 66 | 833 | 0.0022 |
| GO:0006811 | P | ion transport | 39 | 408 | 0.0023 |
| GO:0009064 | P | glutamine family amino acid metabolic process | 10 | 41 | 0.0029 |
| GO:0055085 | P | transmembrane transport | 57 | 701 | 0.0034 |
| GO:0051246 | P | regulation of protein metabolic process | 15 | 106 | 0.012 |
| GO:0005515 | F | protein binding | 188 | 2460 | 4.80E-08 |
| GO:0005215 | F | transporter activity | 91 | 941 | 2.00E-07 |
| GO:0022892 | F | substrate-specific transporter activity | 66 | 593 | 2.50E-07 |
| GO:0022891 | F | substrate-specific transmembrane transporter activity | 58 | 517 | 1.60E-06 |
| GO:0022857 | F | transmembrane transporter activity | 66 | 643 | 2.80E-06 |
| GO:0016798 | F | hydrolase activity, acting on glycosyl bonds | 51 | 480 | 4.00E-05 |
| GO:0016491 | F | oxidoreductase activity | 124 | 1672 | 9.20E-05 |
| GO:0004553 | F | hydrolase activity, hydrolyzing O-glycosyl compounds | 42 | 424 | 0.0016 |
| GO:0048037 | F | cofactor binding | 48 | 543 | 0.0054 |
| GO:0016020 | C | membrane | 151 | 2115 | 5.40E-05 |

P indicates biological process, F indicates molecular function, and C indicates cell component. FDR, false discovery rate.

**Table S6.** Upregulated genes associated with NLRs, RLKs and WRKYs in LM.

| **ID** | **Description** | **log_2_FC** |
| --- | --- | --- |
| **Upregulated genes annotated as NB-ARC or NB-LRR in late meiosis (LM) stage** | | |
| LOC_Os12g10710 | NB-ARC domain containing protein, expressed | 1.42 |
| LOC_Os08g30660 | NB-ARC domain containing protein, expressed | 2.9 |
| LOC_Os08g10260 | NBS-LRR disease resistance protein, putative, expressed | 1.595 |
| LOC_Os06g17930 | NBS-LRR disease resistance protein, putative, expressed | 5.814 |
| LOC_Os01g52330 | NB-ARC domain containing protein, expressed | 4.648 |
| LOC_Os06g48520 | NBS-LRR, disease resistance protein RPM1, putative, expressed | 1.681 |
| LOC_Os07g33690 | NBS-LRR type disease resistance protein Hom-F, putative, expressed | 3.353 |
| LOC_Os07g02570 | NB-ARC domain containing protein, expressed | 3.433 |
| LOC_Os02g35210 | NBS-LRR, resistance protein, putative, expressed | 1.265 |
| LOC_Os01g25740 | NBS-LRR, powdery mildew resistance protein PM3F, putative, expressed | 1.708 |
| LOC_Os09g19280 | NB-ARC, retrotransposon protein, putative, unclassified, expressed | 7.777 |
| LOC_Os11g45970 | NBS-LRR disease resistance protein, putative, expressed | 1.737 |
| LOC_Os07g17250 | NBS-LRR, disease resistance RPP13-like protein 1, putative, expressed | 1.136 |
| LOC_Os02g18000 | NBS-LRR, disease resistance protein RGA2, putative, expressed | 1.312 |
| LOC_Os08g07340 | NBS-LRR, mla1, putative, expressed | 1.284 |
| LOC_Os11g10120 | NB-ARC, expressed protein | 3.503 |
| LOC_Os09g34160 | NBS-LRR, resistance protein, putative, expressed | 2.451 |
| LOC_Os01g52320 | NB-ARC domain containing protein, expressed | 4.904 |
| LOC_Os03g40194 | NBS-LRR, disease resistance RPP13-like protein 1, putative, expressed | 2.737 |
| LOC_Os11g34920 | NBS-LRR, stripe rust resistance protein Yr10, putative, expressed | 2.91 |
| LOC_Os04g30930 | NB-ARC domain containing protein, expressed | 1.418 |
| LOC_Os08g07380 | NB-ARC, retrotransposon protein, putative, unclassified, expressed | 1.022 |
| LOC_Os11g41170 | NB-ARC, disease resistance protein RPM1, putative, expressed | 1.226 |
| LOC_Os01g52340 | NB-ARC domain containing protein, expressed | 2.028 |
| LOC_Os01g16400 | NBS-LRR, NB-ARC domain containing protein, expressed | 3.796 |
| LOC_Os12g25170 | NB-ARC domain containing disease resistance protein, putative, expressed | 1.005 |
| LOC_Os11g37880 | NBS-LRR, stripe rust resistance protein Yr10, putative, expressed | 7.907 |
| LOC_Os12g28100 | NBS-LRR disease resistance protein, putative, expressed | 3.88 |
| LOC_Os02g18070 | NB-ARC domain containing protein, expressed | 2.48 |
| LOC_Os09g14490 | TIR-NBS type disease resistance protein, putative, expressed | 2.397 |
| **Upregulated genes annotated as receptor-like protein kinase in late meiosis (LM) stage** | | |
| LOC_Os07g38810 | lectin receptor-type protein kinase, putative, expressed | 1.564 |
| LOC_Os07g04010 | lectin-like receptor kinase, putative | 6.217 |
| LOC_Os01g60060 | leucine-rich repeat family protein, putative, expressed | 1.042 |
| LOC_Os11g44860 | cysteine-rich receptor-like protein kinase 28 precursor, putative, expressed | 2.825 |
| LOC_Os12g10740 | leucine-rich repeat family protein, putative, expressed | 1.301 |
| LOC_Os11g36150 | receptor-like protein kinase 2 precursor, putative, expressed | 1.699 |
| LOC_Os09g37834 | erine/threonine-protein kinase receptor precursor, putative, expressed | 3.019 |
| LOC_Os01g53920 | receptor-like protein kinase 5 precursor, putative, expressed | 3.462 |
| LOC_Os11g11780 | cysteine-rich receptor-like protein kinase 21 precursor, putative, expressed | 5.51 |
| LOC_Os04g29680 | OsWAK38 - OsWAK receptor-like protein kinase, expressed | 5.183 |
| LOC_Os08g03240 | lectin-like receptor kinase 1, putative, expressed | 4.014 |
| LOC_Os04g29960 | OsWAK43 - OsWAK receptor-like protein kinase, expressed | 6.619 |
| LOC_Os07g03820 | lectin-like receptor kinase, putative, expressed | 5.820 |
| LOC_Os03g56160 | lectin-like receptor kinase 7, putative, expressed | 7.086 |
| LOC_Os05g16740 | SHR5-receptor-like kinase, putative, expressed | 1.914 |
| LOC_Os04g15630 | xa21, putative | 1.323 |
| LOC_Os02g13430 | receptor-like protein kinase 5 precursor, putative, expressed | 3.129 |
| LOC_Os08g24310 | receptor-like protein kinase precursor, putative, expressed | 1.832 |
| LOC_Os07g03810 | lectin-like receptor kinase 7, putative, expressed | 1.800 |
| LOC_Os04g28780 | serine/threonine-protein kinase receptor precursor, putative, expressed | 2.898 |
| LOC_Os08g10330 | SHR5-receptor-like kinase, putative, expressed | 1.312 |
| LOC_Os02g12030 | receptor-like protein kinase precursor, putative | 6.500 |
| LOC_Os06g36320 | receptor-like protein kinase 5 precursor, putative, expressed | 1.103 |
| LOC_Os08g03170 | lectin-like receptor kinase 1, putative, expressed | 3.136 |
| LOC_Os11g40970 | receptor-like protein kinase precursor, putative, expressed | 7.628 |
| LOC_Os02g12420 | receptor-like protein kinase precursor, putative, expressed | 6.832 |
| LOC_Os06g47700 | serine/threonine-protein kinase BRI1-like 2 precursor, putative, expressed | 2.142 |
| LOC_Os07g03790 | lectin-like receptor kinase 7, putative, expressed | 6.741 |
| LOC_Os09g38834 | OsWAK89b - OsWAK receptor-like protein kinase | 7.667 |
| LOC_Os02g11980 | receptor-like protein kinase precursor, putative, expressed | 2.001 |
| LOC_Os02g13510 | receptor-like protein kinase 5 precursor, putative, expressed | 2.687 |
| LOC_Os10g02970 | Leucine rich repeat N-terminal domain containing protein, putative, expressed | 1.938 |
| LOC_Os10g02360 | OsWAK98 - OsWAK receptor-like cytoplasmic kinase OsWAK-RLCK, expressed | 3.843 |
| LOC_Os07g03780 | lectin-like receptor kinase, putative | 3.526 |
| LOC_Os08g13420 | S-domain receptor-like protein kinase, putative | 6.141 |
| LOC_Os11g35120 | OsWAK116 - OsWAK receptor-like cytoplasmic kinase OsWAK-RLCK | 5.262 |
| LOC_Os09g38840 | OsWAK90 - OsWAK receptor-like protein kinase, expressed | 6.379 |
| LOC_Os04g30010 | OsWAK45 - OsWAK receptor-like protein kinase, expressed | 1.797 |
| LOC_Os08g10310 | SHR5-receptor-like kinase, putative, expressed | 3.374 |
| LOC_Os07g31190 | OsWAK71 - OsWAK receptor-like cytoplasmic kinase OsWAK-RLCK, expressed | 2.571 |
| LOC_Os05g44770 | receptor-like protein kinase 5 precursor, putative, expressed | 3.209 |
| LOC_Os08g25380 | serine/threonine-protein kinase BRI1-like 1 precursor, putative, expressed | 2.496 |
| LOC_Os04g20680 | wall-associated receptor kinase 3 precursor, putative, expressed | 3.655 |
| LOC_Os04g30160 | OsWAK46 - OsWAK receptor-like protein kinase, expressed | 3.655 |
| LOC_Os08g10300 | SHR5-receptor-like kinase, putative, expressed | 6.425 |
| LOC_Os12g43640 | receptor-like protein kinase HAIKU2 precursor, putative, expressed | 1.749 |
| LOC_Os11g36140 | receptor-like protein kinase 2 precursor, putative, expressed | 3.455 |
| **Upregulated genes annotated as WRKYs protein kinase in late meiosis (LM) stage** | | |
| LOC_Os01g40260 | WRKY77, expressed | 6.26 |
| LOC_Os05g45230 | WRKY58, expressed | 4.65 |
| LOC_Os05g39720 | WRKY70, expressed | 4.64 |
| LOC_Os09g25070 | WRKY62, expressed | 4.63 |
| LOC_Os11g29870 | WRKY72, expressed | 4.17 |
| LOC_Os05g49100 | WRKY49, expressed | 3.86 |
| LOC_Os01g61080 | WRKY24, expressed | 3.56 |
| LOC_Os05g04640 | WRKY5, expressed | 3.51 |
| LOC_Os05g25770 | WRKY45, expressed | 2.94 |
| LOC_Os05g46020 | WRKY7, expressed | 2.83 |
| LOC_Os01g14440 | WRKY1, expressed | 2.45 |
| LOC_Os01g53260 | WRKY23, expressed | 2.30 |
| LOC_Os09g16510 | WRKY74, expressed | 2.03 |
| LOC_Os01g09080 | WRKY107, expressed | 1.66 |
| LOC_Os01g53040 | WRKY14, expressed | 1.43 |
| LOC_Os03g20550 | WRKY55, expressed | 1.37 |
| LOC_Os08g29660 | WRKY69, expressed | 1.24 |
| LOC_Os03g55080 | WRKY3, expressed | 1.23 |
| LOC_Os05g09020 | WRKY67, expressed | 1.20 |
| LOC_Os01g18584 | WRKY9, expressed | 1.16 |
| LOC_Os01g54600 | WRKY13, expressed | 1.01 |

**Table S7.** DEGs associated with pathogenesis-related proteins in the EM and LM.

| **PR family** | **Gene** | **ID** | **Annotation** | **EM log_2_FC** | **LM log_2_FC** |
| --- | --- | --- | --- | --- | --- |
| PR3 | *OsCHIT7* | LOC_Os06g51050 | chitinase | 4.02 | 0.04 |
|  | *OsCHIT8* | LOC_Os06g51060 | Chitinase family protein precursor, expressed | 4.05 | 1.99 |
|  | *OsCHIT14* | LOC_Os10g39680 | Chitinase family protein precursor, expressed | 1.21 | 2.37 |
|  | *OsCHIT16* | LOC_Os03g04060 | chitinase gene | 1.56 | 4.62 |
|  | *OsCHIT17* | LOC_Os05g33130 | Chitinase family protein precursor, expressed | 1.27 | 0.96 |
| PR4 | *OsPR4a* | LOC_Os11g37970 | Wound-induced protein precursor, expressed | 3.87 | 3.47 |
|  | *OsPR4b* | LOC_Os11g37960 | Wound-induced protein precursor, expressed | 3 | 6.16 |
|  | *OsPR4c* | LOC_Os11g37950 | Wound-induced protein precursor, expressed | 3.33 | 6.46 |
|  | *OsPR4d* | LOC_Os11g37940 | Wound-induced protein precursor, expressed | 3.94 | 6.13 |
| PR5 | *TLP* | LOC_Os03g46070 | thaumatin, putative, expressed | 3.03 | 4.29 |
|  | *PR5K* | LOC_Os03g14050 | thaumatin-like protein 1 precursor, putative, expressed | 0.15 | 1.07 |
| PR8 | *Gns11* | LOC_Os07g35480 | glucan endo-1,3-beta-glucosidase precursor, putative, expressed | 0.75 | 0.98 |
|  | *Gns12* | LOC_Os07g35520 | glucan endo-1,3-beta-glucosidase precursor, putative, expressed | -0.04 | 0.37 |
|  | *Gns13* | LOC_Os07g35510 | glucan endo-1,3-beta-glucosidase precursor, putative, expressed | -1.66 | 2.04 |
|  | *Gns14* | LOC_Os07g35350 | glucan endo-1,3-beta-glucosidase precursor, putative, expressed | 4.16 | 2.04 |
|  | *Oschib1* | LOC_Os10g28080 | glycosyl hydrolase, putative, expressed | 3.99 | 5.03 |
| PR10 | *PR-10a* | LOC_Os12g36880 | Orysa sativa pathogenesis-related protein 10a | 5.09 | 5.07 |
|  | *PR-10b* | LOC_Os12g36850 | pathogenesis-related Bet v I family protein, putative, expressed | 3.73 | 3.07 |
|  | *PR-10c* | LOC_Os03g18850 | jasmonate inducible rice PR10 | 1.93 | 0.45 |
|  | *RSOsPR10* | LOC_Os12g36830 | root specific rice PR10 | 2.82 | 4.48 |
| PR13 | *Osthi12* | LOC_Os06g32600 | THION15 - Plant thionin family protein precursor, expressed | -0.08 | 1.55 |
| PR14 | *RLTP2* | LOC_Os03g02050 | Protease inhibitor/seed storage/LTP family protein precursor, expressed | 0.23 | -2.56 |
|  | *LTP110* | LOC_Os11g02350 | Protease inhibitor/seed storage/LTP family protein precursor, expressed | -0.09 | 0.34 |
|  | *b21* | LOC_Os12g02300 | Protease inhibitor/seed storage/LTP family protein precursor, expressed | 0.02 | 0.4 |
|  | *OsLTP2* | LOC_Os12g02320 | Protease inhibitor/seed storage/LTP family protein precursor, expressed | -0.31 | 0.6 |
|  | *OsLTP5* | LOC_Os11g02389 | protease inhibitor/seed storage/LTP family, putative, expressed | -1.53 | 1.19 |
| PR16 | *OsGLP8-3* | LOC_Os08g08970 | Cupin domain containing protein; germin-like protein | 1.86 | 3.39 |
|  | *OsGLP8-4* | LOC_Os08g08980 | Germin-Like Protein | 2.04 | 0.93 |
|  | *OsGLP8-5* | LOC_Os08g08990 | Cupin domain containing protein, expressed | - | 0.84 |
|  | *OsGLP8-6* | LOC_Os08g09000 | Cupin domain containing protein, Germin-like protein | 5.67 | 4.36 |
|  | *OsGLP8-7* | LOC_Os08g09010 | Cupin domain containing protein, Germin-like protein | 8.11 | 7.65 |
|  | *OsGLP8-8* | LOC_Os08g09020 | Cupin domain containing protein, Germin-like protein | - | 7.14 |
|  | *OsGLP8-9* | LOC_Os08g09040 | Cupin domain containing protein, Germin-like protein | - | 7.14 |
|  | *OsGLP8-10* | LOC_Os08g09060 | Cupin domain containing protein, Germin-like protein | - | 5.99 |

**Table S8.** DEGs related to biotic stress and protein degradation based on MapMan analysis.

| **ID** | **MMC log_2_FC** | **EM log_2_FC** | **LM log_2_FC** | **MP log_2_FC** |
| --- | --- | --- | --- | --- |
| **NB-LRR** | | | | |
| LOC_Os01g16400 | 0.28147 | 0.670889 | 3.795734 | 2.169416 |
| LOC_Os01g25740 | -0.07848 | -0.11622 | 1.707571 | 1.03093 |
| LOC_Os01g52320 | - | 5.107121 | 4.904509 | -1.85165 |
| LOC_Os01g52330 | -0.84754 | 3.607133 | 4.647662 | 0.7496 |
| LOC_Os01g52340 | 0.149929 | -0.28466 | 2.027959 | -0.08564 |
| LOC_Os02g18000 | 0.353827 | 0.321891 | 1.311723 | 0.450467 |
| LOC_Os02g18070 | 1.099895 | 3.820908 | 2.479727 | 1.555634 |
| LOC_Os02g35210 | 0.410898 | 0.439302 | 1.264557 | 0.815425 |
| LOC_Os03g40194 | 0.691552 | 0.672982 | 2.737176 | 0.209914 |
| LOC_Os04g30930 | 0.191436 | 0.323298 | 1.418413 | 0.113933 |
| LOC_Os06g17930 | - | 2.007135 | 5.814007 | -2.96134 |
| LOC_Os06g48520 | 0.19712 | 0.456178 | 1.681112 | -0.2479 |
| LOC_Os07g02570 | 0.458715 | 2.072047 | 3.433287 | 1.150879 |
| LOC_Os07g17250 | 0.065118 | 0.527512 | 1.135831 | 0.895599 |
| LOC_Os07g33690 | 0.198345 | 2.184259 | 3.353436 | -1.00596 |
| LOC_Os08g07340 | 0.535364 | 0.347313 | 1.283614 | 0.245274 |
| LOC_Os08g07380 | 0.257948 | 0.177483 | 1.021852 | 0.723659 |
| LOC_Os08g10260 | 1.797596 | 2.358063 | 1.59525 | 2.024746 |
| LOC_Os08g30660 | -1.21525 | 3.417604 | 2.900341 | 3.766459 |
| LOC_Os09g14490 | 0.570238 | 1.003963 | 2.397205 | 0.364005 |
| LOC_Os09g19280 | 0.936446 | 5.532426 | 7.77704 | -0.89188 |
| LOC_Os09g34160 | 0.409592 | -0.11334 | 2.45114 | -0.40228 |
| LOC_Os11g10120 | 0.860913 | 0.570214 | 3.503302 | -1.67519 |
| LOC_Os11g34920 | 0.219469 | 0.26552 | 2.90972 | 0.213903 |
| LOC_Os11g37880 | -0.09102 | 1.783341 | 7.90708 | 2.668009 |
| LOC_Os11g41170 | 0.087306 | 0.578887 | 1.226506 | 1.142148 |
| LOC_Os11g45970 | 0.348469 | 0.228318 | 1.737024 | 0.301013 |
| LOC_Os12g10710 | 0.108081 | 0.301887 | 1.420335 | 0.571126 |
| LOC_Os12g25170 | 0.321743 | 0.198928 | 1.005099 | 1.274974 |
| LOC_Os12g28100 | 0.236053 | 0.931639 | 3.880076 | -1.65581 |
| **PR-proteins** | | | | |
| LOC_Os06g51050 | -0.82416 | 4.002994 | 0.023454 | -0.21602 |
| LOC_Os06g51060 | 0.224364 | 4.036562 | 1.983507 | 0.039345 |
| LOC_Os10g39680 | 1.008393 | 1.194013 | 2.36326 | -0.61317 |
| LOC_Os03g04060 | -1.15336 | 1.539203 | 4.610544 | -0.44662 |
| LOC_Os05g33130 | -0.24856 | 1.270955 | 0.952097 | 0.932821 |
| LOC_Os11g37970 | 1.16612 | 3.858273 | 3.445913 | 0.903884 |
| LOC_Os11g37960 | 0.576695 | 2.985442 | 6.09741 | 0.370808 |
| LOC_Os11g37950 | -0.74122 | 3.319276 | 6.445929 | 0.204247 |
| LOC_Os11g37940 | 1.65153 | 3.90908 | 6.06267 | 1.651496 |
| LOC_Os03g46070 | -0.76604 | 3.022324 | 4.242381 | -1.22127 |
| LOC_Os03g14050 | -0.15701 | 0.157787 | 1.066813 | 1.217999 |
| LOC_Os07g35480 | -0.77245 | 0.736147 | 0.965922 | -2.00087 |
| LOC_Os07g35520 | 0.687378 | -0.03089 | 0.365738 | -1.70898 |
| LOC_Os07g35510 | -0.67663 | -1.6713 | 2.033374 | -0.56218 |
| LOC_Os07g35350 | -0.99121 | 4.149626 | 2.011029 | -0.20496 |
| LOC_Os10g28080 | -0.01023 | 3.968061 | 4.981346 | -0.1709 |
| LOC_Os12g36880 | -1.13151 | 5.082608 | 5.057996 | -0.09203 |
| LOC_Os12g36850 | -0.68788 | 3.716498 | 3.063296 | -0.68176 |
| LOC_Os03g18850 | 0.664339 | 1.923357 | 0.444327 | -0.97575 |
| LOC_Os12g36830 | -0.38651 | 2.808095 | 4.473404 | 0.09601 |
| LOC_Os06g32600 | 0.582334 | -0.07112 | 1.540193 | 0.338443 |
| LOC_Os03g02050 | 1.678966 | 0.21384 | -2.56489 | 2.201359 |
| LOC_Os11g02350 | 0.401304 | -0.0806 | 0.328226 | 0.351928 |
| LOC_Os12g02300 | 0.610109 | 0.025089 | 0.388404 | -0.28828 |
| LOC_Os12g02320 | -0.09517 | -0.30814 | 0.593806 | 0.027147 |
| LOC_Os11g02389 | -0.81647 | -1.54086 | 1.18206 | 1.387956 |
| LOC_Os08g08970 | -4.00405 | 1.846096 | 3.376566 | 1.597101 |
| LOC_Os08g08980 | -0.16009 | 2.020727 | 0.91814 | 0.077476 |
| LOC_Os08g08990 | 0.066121 | 3.21539 | 0.837555 | -0.32598 |
| LOC_Os08g09000 | -0.29801 | 5.637785 | 4.353354 | -1.07756 |
| LOC_Os08g09010 | -0.4346 | 8.108756 | 7.618606 | -0.37356 |
| LOC_Os08g09020 | - | 4.628427 | 7.136867 | -1.64725 |
| LOC_Os08g09040 | 2.671429 | 5.946615 | 8.387693 | -0.84149 |
| LOC_Os08g09060 | - | 6.086189 | 5.991433 | -0.31634 |
| **Receptor like protein kinase** | | | | |
| LOC_Os01g53920 | 1.041165 | 0.527385 | 3.461883 | -1.35129 |
| LOC_Os01g60060 | 0.108322 | 0.29097 | 1.043019 | 0.136898 |
| LOC_Os02g11980 | 0.057661 | 0.41179 | 2.00119 | -0.63886 |
| LOC_Os02g12030 | 1.759544 | 1.221891 | 6.500279 | -1.00516 |
| LOC_Os02g12420 | 1.545497 | 1.776757 | 6.831824 | -0.29181 |
| LOC_Os02g13430 | -0.38321 | 1.333069 | 3.128917 | -0.14198 |
| LOC_Os02g13510 | -0.63775 | -0.40777 | 2.687506 | -0.31474 |
| LOC_Os03g56160 | 2.197444 | 2.762901 | 7.086082 | 0.966027 |
| LOC_Os04g15630 | 0.22931 | 1.301945 | 1.323148 | -0.04715 |
| LOC_Os04g20680 | 0.287261 | 4.688146 | 3.655031 | 0.234442 |
| LOC_Os04g28780 | 2.57862 | 4.438422 | 2.897779 | 1.881887 |
| LOC_Os04g29680 | 0.021186 | 1.291105 | 5.182575 | 0.550114 |
| LOC_Os04g29960 | 0.157899 | -1.24465 | 6.6195 | -2.78523 |
| LOC_Os04g30010 | 0.007675 | -0.12926 | 1.796846 | -1.10326 |
| LOC_Os04g30160 | -0.07399 | 0.808614 | 6.424883 | -1.58354 |
| LOC_Os05g16740 | -0.08101 | 1.177758 | 1.914475 | 1.356391 |
| LOC_Os05g44770 | 0.587429 | 0.684289 | 3.208606 | -1.68843 |
| LOC_Os06g36320 | -0.06181 | 1.152894 | 1.103505 | -1.09894 |
| LOC_Os06g47700 | -1.23709 | 1.754451 | 2.141723 | -1.66483 |
| LOC_Os07g03780 | 0.302892 | 1.310916 | 3.525933 | 0.033798 |
| LOC_Os07g03790 | - | 2.622456 | 6.741445 | 1.574241 |
| LOC_Os07g03810 | 0.180165 | 0.277934 | 1.800099 | 0.850136 |
| LOC_Os07g03820 | 0.585197 | 4.188537 | 5.820471 | 1.695694 |
| LOC_Os07g04010 | -0.1051 | 1.400367 | 6.217268 | -0.53247 |
| LOC_Os07g31190 | -0.6583 | 1.565853 | 2.571841 | 0.887262 |
| LOC_Os07g38810 | 0.307231 | 0.740971 | 1.56458 | 0.232547 |
| LOC_Os08g03170 | -0.56154 | -1.4925 | 3.13617 | - |
| LOC_Os08g03240 | 1.67158 | 5.08843 | 4.013849 | -0.90481 |
| LOC_Os08g10300 | 0.509134 | 0.154502 | 1.235327 | -0.18172 |
| LOC_Os08g10310 | 0.133528 | 2.359152 | 3.374063 | 0.381219 |
| LOC_Os08g10330 | 0.008971 | 0.186392 | 1.312135 | 0.197099 |
| LOC_Os08g13420 | - | - | 6.141316 | 0.403925 |
| LOC_Os08g24310 | 0.798581 | 0.752123 | 1.831817 | -0.73828 |
| LOC_Os08g25380 | 0.396761 | 0.549559 | 2.495799 | 0.899638 |
| LOC_Os09g37834 | 0.225653 | 3.040572 | 3.019658 | -1.59716 |
| LOC_Os09g38834 | -1.14499 | 1.80359 | 7.667506 | 0.438177 |
| LOC_Os09g38840 | 1.167176 | 1.463742 | 6.379358 | 2.39968 |
| LOC_Os10g02360 | -0.16863 | 0.552587 | 3.843362 | -0.67978 |
| LOC_Os10g02970 | -0.026 | 0.313707 | 1.937807 | 1.880796 |
| LOC_Os11g11780 | 1.982618 | -2.80243 | 5.509939 | -2.40874 |
| LOC_Os11g35120 | -0.0968 | 4.096135 | 5.262293 | -2.10976 |
| LOC_Os11g36140 | -0.37013 | 1.21135 | 3.454993 | 0.418956 |
| LOC_Os11g36150 | -0.25362 | 0.507374 | 1.698587 | -0.83156 |
| LOC_Os11g40970 | -0.10424 | 2.881723 | 7.627707 | -1.03751 |
| LOC_Os11g44860 | 0.147372 | 1.385388 | 2.825335 | -0.16778 |
| LOC_Os12g10740 | 0.613569 | 0.446574 | 1.301516 | -0.69297 |
| LOC_Os12g43640 | 0.28038 | -0.00509 | 1.748748 | -1.28567 |
| ***WRKY*** | | | | |
| LOC_Os01g09080 | 1.164286 | -1.05363 | 1.663058 | 0.954009 |
| LOC_Os01g14440 | 0.700805 | 0.483609 | 2.454944 | -0.5404 |
| LOC_Os01g18584 | 0.143996 | 0.931639 | 1.16451 | 0.26901 |
| LOC_Os01g40260 | 0.007939 | 1.331055 | 6.258033 | -0.68574 |
| LOC_Os01g53040 | -1.30182 | -0.00545 | 1.430325 | 0.868228 |
| LOC_Os01g53260 | - | 5.208145 | 2.300464 | -5.39847 |
| LOC_Os01g54600 | -0.38367 | -0.6192 | 1.015009 | 0.637839 |
| LOC_Os01g61080 | 0.461121 | 0.217772 | 3.558065 | -1.01398 |
| LOC_Os03g20550 | 0.198399 | 0.404105 | 1.370655 | 0.296575 |
| LOC_Os03g55080 | -0.07428 | 1.539249 | 1.227826 | 0.691157 |
| LOC_Os05g04640 | -0.72599 | 2.062314 | 3.513894 | -0.67278 |
| LOC_Os05g09020 | 1.643806 | 0.150244 | 1.203736 | 1.073477 |
| LOC_Os05g25770 | -0.26344 | 1.224845 | 2.939275 | -0.40566 |
| LOC_Os05g39720 | 0.433819 | -0.02263 | 4.640428 | -1.89447 |
| LOC_Os05g45230 | - | 7.054295 | 4.649715 | - |
| LOC_Os05g46020 | 0.732128 | 0.502429 | 2.827722 | -0.37933 |
| LOC_Os05g49100 | 0.55566 | 0.88817 | 3.861159 | 0.221502 |
| LOC_Os08g29660 | 0.642823 | 0.474636 | 1.24206 | -0.33622 |
| LOC_Os09g16510 | 0.124689 | 1.013603 | 2.028043 | -0.10092 |
| LOC_Os09g25070 | 0.731987 | 2.329737 | 4.628894 | -4.05081 |
| LOC_Os11g29870 | -0.74796 | 0.703919 | 4.172476 | -0.13897 |
| ***ERF*** | | | | |
| LOC_Os02g40070 | -1.16044 | -0.77796 | -5.16224 | - |
| LOC_Os04g32620 | -0.24437 | -2.21071 | 1.511326 | 0.699217 |
| LOC_Os04g37520 | 1.585938 | 0.061946 | -4.16637 | -4.1828 |
| LOC_Os06g06540 | - | 0.196097 | -8.79816 | - |
| LOC_Os08g34360 | -0.61679 | 0.59915 | -3.18607 | -0.22547 |
| LOC_Os09g25600 | -0.00138 | -0.63633 | -1.60694 | -0.31974 |
| LOC_Os11g03540 | 0.113905 | 1.117199 | -1.94394 | -1.29105 |
| LOC_Os12g03290 | 0.549262 | 0.159391 | -1.88699 | -3.29904 |
| ***bZIP*** | | | | |
| LOC_Os01g05480 | 0.067304 | -0.31155 | -3.83024 | 0.198607 |
| LOC_Os01g08990 | 1.821943 | -0.37026 | -6.23874 | -5.24309 |
| LOC_Os01g64020 | -0.54663 | -0.12702 | -1.00533 | -2.3562 |
| LOC_Os02g03960 | 0.810526 | 0.147217 | -1.19935 | -1.24333 |
| LOC_Os03g08960 | -0.25604 | -0.09306 | 1.011457 | 0.971364 |
| LOC_Os03g10320 | -0.78758 | -0.97878 | -4.43388 | -0.43498 |
| LOC_Os05g37170 | -0.26241 | 0.169208 | -1.10317 | -3.38207 |
| LOC_Os05g41280 | -0.16746 | -0.47563 | -7.43369 | 1.505541 |
| LOC_Os06g41100 | -0.26188 | 1.058358 | 1.065165 | -0.2749 |
| LOC_Os07g39320 | -0.8024 | 1.06714 | 1.587583 | -0.44046 |
| LOC_Os09g10840 | -0.80952 | 3.896389 | 1.181101 | 0.013009 |
| LOC_Os09g21180 | -1.03943 | -2.11997 | -2.10129 | -2.204 |
| LOC_Os09g21380 | -0.92554 | -0.86142 | -1.8996 | -0.74844 |
| LOC_Os09g31390 | 0.193889 | -0.31392 | -1.2473 | -1.58297 |
| LOC_Os11g05480 | 0.377162 | -0.57024 | -1.65014 | -1.71439 |
| LOC_Os12g40920 | 0.291138 | 0.720673 | 1.101654 | 2.162375 |
| ***MYB*** | | | | |
| LOC_Os01g03720 | -0.6559 | -0.15483 | 5.725969 | 2.12732 |
| LOC_Os01g16810 | -0.17722 | -1.08299 | -3.2269 | -0.54986 |
| LOC_Os01g32574 | - | - | -6.5793 | -3.32971 |
| LOC_Os01g45090 | -0.60268 | 0.138866 | 2.446663 | 1.232222 |
| LOC_Os01g49160 | 0.43457 | -0.84133 | -1.48654 | -0.94393 |
| LOC_Os01g51260 | -0.56985 | -0.14461 | -2.45559 | 0.536114 |
| LOC_Os02g46030 | -0.25145 | 1.069826 | -5.59135 | -3.88766 |
| LOC_Os02g46780 | -0.2259 | 1.120561 | 3.155207 | -0.18411 |
| LOC_Os02g49250 | - | -1.2871 | -3.31817 | -2.75141 |
| LOC_Os03g13310 | -0.04334 | -0.52074 | -1.89017 | -0.24692 |
| LOC_Os03g18480 | -2.06149 | -0.84552 | -3.57897 | - |
| LOC_Os03g38210 | -0.27076 | -0.54489 | -7.11386 | 1.363619 |
| LOC_Os04g39470 | -1.63676 | -1.3603 | -3.68926 | 0.824868 |
| LOC_Os04g45020 | -0.37212 | -0.88645 | -1.64938 | -2.38329 |
| LOC_Os04g50680 | 1.65153 | 5.991017 | 6.013901 | 1.669465 |
| LOC_Os05g18880 | 1.661011 | -2.25736 | -4.70904 | -1.35182 |
| LOC_Os05g28320 | -3.61047 | -0.78242 | -1.08628 | -4.32445 |
| LOC_Os05g38890 | -0.88364 | -0.83115 | -1.45422 | -5.80994 |
| LOC_Os06g02250 | -0.62459 | 5.977647 | 4.40316 | -0.35018 |
| LOC_Os06g14670 | -0.79626 | -0.332 | -3.91694 | -0.10057 |
| LOC_Os06g18780 | -0.51227 | -0.41275 | 1.322216 | -2.31323 |
| LOC_Os06g45890 | -0.3158 | 2.128269 | 3.664589 | 0.154289 |
| LOC_Os07g44030 | -0.67476 | 0.288605 | -1.0782 | -2.92868 |
| LOC_Os07g48870 | -0.51709 | 0.371651 | 3.276835 | -0.42307 |
| LOC_Os08g08680 | 0.818575 | 0.620725 | 1.267693 | 1.40744 |
| LOC_Os08g26010 | - | 1.633064 | 6.501669 | -2.23844 |
| LOC_Os08g33150 | -0.43904 | 0.91577 | -5.8204 | 1.432989 |
| LOC_Os09g23620 | 0.681707 | -0.26692 | -7.30224 | -0.97748 |
| LOC_Os10g08450 | -1.41566 | -0.58735 | -9.00188 | -3.80749 |
| LOC_Os10g13430 | 0.218357 | -0.03344 | -1.47264 | -3.8861 |
| LOC_Os11g01480 | -0.74825 | 0.102399 | 1.16471 | 0.665666 |
| LOC_Os11g10130 | - | 1.149576 | 1.995334 | 1.166965 |
| LOC_Os11g45740 | -0.01283 | -0.46861 | 2.840454 | 1.122833 |
| LOC_Os12g01490 | -0.33786 | 0.550568 | 1.109685 | 1.515794 |
| LOC_Os12g07640 | -0.69557 | 1.858979 | 2.054193 | 1.088258 |
| LOC_Os12g09420 | 0.143996 | -1.24465 | -3.51797 | -2.63768 |
| LOC_Os12g23520 | 0.111971 | 0.440167 | -9.89108 | 1.032131 |
| **Cell wall** | | | | |
| LOC_Os05g51670 | 0.061477 | -0.39487 | -1.70952 | -0.9185 |
| LOC_Os09g35800 | -0.28453 | 0.244148 | -2.78337 | -0.72962 |
| LOC_Os03g31210 | - | 0.153626 | -5.28963 | -7.2096 |
| LOC_Os01g03710 | -0.07242 | -0.22082 | -1.45212 | -1.42853 |
| LOC_Os09g22090 | 0.594913 | -0.89893 | -5.97286 | -3.17259 |
| LOC_Os11g38810 | 0.105358 | -0.32569 | -2.65984 | -2.07628 |
| LOC_Os04g35030 | -1.26864 | - | -7.02413 | -2.10976 |
| LOC_Os06g42020 | -0.39134 | 0.008027 | -1.76075 | 1.194665 |
| LOC_Os08g25710 | 1.801163 | -1.87343 | -3.99614 | -11.1024 |
| LOC_Os09g30120 | -0.12083 | 0.320882 | 3.417564 | -0.5263 |
| LOC_Os09g30130 | -1.30175 | 0.39267 | 1.442573 | -0.70758 |
| LOC_Os07g41310 | 0.878494 | 0.45668 | -2.73245 | -0.56269 |
| LOC_Os01g06580 | -0.31227 | -1.60511 | -1.35379 | -0.93549 |
| LOC_Os02g49420 | 0.138573 | -1.27617 | -1.30642 | -1.83154 |
| LOC_Os05g07060 | - | -1.24465 | -3.82292 | -2.78068 |
| LOC_Os06g44660 | 1.65153 | -2.2187 | -11.3058891 | -10.4700573 |
| LOC_Os06g10910 | 1.545497 | - | -6.00441 | -8.10412 |
| LOC_Os10g05750 | -0.1951 | -0.21234 | -2.54307 | -2.3221 |
| LOC_Os10g05950 | 0.260726 | -0.80534 | -1.1493 | -0.51095 |
| LOC_Os04g48380 | -0.99557 | -0.23886 | -1.76904 | -2.55549 |
| LOC_Os07g40740 | -1.26558 | -0.19393 | -8.71771 | -2.78753 |
| LOC_Os03g53790 | 0.254803 | -0.76451 | -8.064 | -3.2957 |
| LOC_Os06g14540 | -0.59399 | 0.319663 | -1.11026 | -0.70558 |
| LOC_Os08g29770 | -0.07273 | -0.69113 | -4.52995 | -3.61547 |
| LOC_Os08g32940 | - | - | -6.17319 | 1.032131 |
| LOC_Os01g04290 | -0.44469 | -3.28298 | -7.61629 | -1.41649 |
| LOC_Os02g52800 | -1.41566 | - | -7.97041 | 0.899688 |
| LOC_Os03g10440 | -0.47414 | -0.13323 | 1.920192 | 0.248768 |
| LOC_Os03g10478 | -0.22007 | -0.02365 | -7.07951 | -2.7825 |
| LOC_Os03g20420 | -0.26819 | -0.06473 | 2.667552 | 0.547834 |
| LOC_Os05g23830 | 0.932116 | - | -7.64144 | -1.88759 |
| LOC_Os06g22919 | -0.00908 | 0.596504 | 2.678884 | -0.6975 |
| LOC_Os06g48180 | -1.25183 | -2.00113 | -1.73443 | 0.747385 |
| LOC_Os06g48200 | 0.178281 | -0.70668 | -1.16267 | 0.455964 |
| LOC_Os11g47350 | -1.26864 | -1.21432 | -7.80611 | -2.10976 |
| LOC_Os01g66710 | 2.395214 | -1.34449 | -3.67219 | - |
| LOC_Os02g03750 | -0.45122 | -0.98679 | -1.39835 | -4.42334 |
| LOC_Os03g11760 | 1.787576 | -0.71547 | -1.9486 | - |
| LOC_Os05g50260 | -0.06082 | -0.21212 | 1.244588 | -0.56902 |
| LOC_Os06g40880 | -3.49239 | -0.90392 | -11.8249156 | - |
| LOC_Os09g16010 | -2.4429 | -1.23121 | -3.94944 | 0.899688 |
| LOC_Os11g03940 | -1.33328 | 0.296423 | -3.78344 | -1.90795 |
| LOC_Os12g03790 | -1.41566 | -1.34232 | -6.65267 | -3.5899 |
| LOC_Os02g42650 | -0.10913 | -0.12453 | 1.3269 | -2.0691 |
| LOC_Os02g44108 | 0.106039 | 0.647009 | 1.221732 | 2.374999 |
| LOC_Os03g01260 | -0.20923 | -1.03978 | -1.24541 | 0.618066 |
| LOC_Os03g04020 | -0.35723 | -0.03656 | 1.645976 | 0.580524 |
| LOC_Os04g46630 | - | -1.24465 | -6.42437 | -6.01324 |
| LOC_Os06g13040 | - | - | -6.44516 | -4.68246 |
| LOC_Os07g29750 | -0.658 | -0.69272 | -1.13486 | 1.942832 |
| LOC_Os10g40700 | -0.22073 | -0.23746 | 2.145815 | -0.08225 |
| LOC_Os10g40710 | -0.82373 | -3.21681 | 3.757917 | -8.38838 |
| LOC_Os10g40720 | 0.098289 | -0.04893 | 1.506364 | -0.32737 |
| LOC_Os01g65790 | -0.23977 | -0.49422 | -1.2974 | -1.29115 |
| LOC_Os07g41650 | -0.61718 | -0.45824 | -3.84059 | -1.15365 |
| LOC_Os08g34910 | -0.28206 | -0.28496 | -3.27685 | -10.3092513 |
| LOC_Os01g66830 | 0.411858 | -0.49332 | -2.62706 | -0.03771 |
| LOC_Os02g47400 | -0.12017 | -1.00426 | -1.68166 | -0.31789 |
| LOC_Os03g43060 | 0.332935 | -0.15993 | -1.85063 | 0.108201 |
| LOC_Os03g44920 | -1.26864 | -2.2187 | -2.27634 | -5.50851 |
| LOC_Os03g46140 | - | - | -4.87524 | - |
| LOC_Os03g46530 | -1.16821 | -1.24465 | -3.29909 | - |
| LOC_Os03g46690 | 1.65153 | 0.198488 | -6.77654 | -8.63114 |
| LOC_Os03g46720 | - | -2.2187 | -8.2114 | -1.88759 |
| LOC_Os03g47420 | 1.964416 | 1.584063 | -1.98663 | -1.88759 |
| LOC_Os07g09110 | 0.322097 | -0.01378 | -13.572484 | -9.1631 |
| **E3 ubiquitin ligase BTB/POZ** | | | | |
| LOC_Os02g20690 | -1.24685 | - | -8.57183 | - |
| LOC_Os02g38320 | -0.06497 | -0.50572 | -1.40499 | -0.31284 |
| LOC_Os04g53390 | - | - | -5.79601 | -2.10976 |
| LOC_Os04g53400 | - | - | -6.55578 | - |
| LOC_Os04g53430 | -0.00304 | -1.41352 | -4.69079 | -0.35018 |
| LOC_Os04g55000 | - | - | -4.22741 | - |
| LOC_Os04g56460 | - | - | -6.60461 | - |
| LOC_Os06g45720 | - | - | -7.76111 | - |
| LOC_Os08g03470 | - | 1.6265 | -6.65095 | -6.04682 |
| LOC_Os08g03480 | - | 0.153626 | -10.4248 | -4.32213 |
| LOC_Os08g03510 | 0.976635 | -0.55685 | -4.77443 | 0.899688 |
| LOC_Os08g13000 | - | -1.3838 | -6.5437 | -7.99844 |
| LOC_Os08g13030 | 1.365566 | 0.06639 | -4.53712 | -1.48622 |
| LOC_Os08g13070 | 1.04878 | -1.60096 | -4.70823 | -4.43377 |
| LOC_Os08g13180 | - | - | -6.34893 | -2.10976 |
| LOC_Os08g13250 | - | 1.633064 | -6.69599 | 1.032131 |
| LOC_Os08g25240 | -1.26864 | -0.85444 | -6.39059 | - |
| LOC_Os08g38700 | -0.75826 | 0.014225 | 2.457833 | 1.082868 |
| LOC_Os08g41120 | - | - | -7.45017 | - |
| LOC_Os08g41170 | - | - | -5.46366 | - |
| LOC_Os08g41190 | - | - | -8.39627 | - |
| LOC_Os08g41240 | - | - | -4.88831 | - |
| LOC_Os09g16850 | - | - | -4.49423 | - |
| LOC_Os10g28760 | - | -1.24465 | -4.11942 | - |
| LOC_Os10g28770 | - | - | -3.21883 | - |
| LOC_Os10g28810 | -1.41566 | 1.196953 | -4.3477 | -0.2528 |
| LOC_Os10g28820 | - | - | -6.75224 | - |
| LOC_Os10g28840 | - | - | -5.79347 | - |
| LOC_Os10g29100 | - | - | -9.31283 | - |
| LOC_Os10g29120 | - | -1.24465 | -5.66389 | - |
| LOC_Os10g29150 | - | - | -6.23816 | - |
| LOC_Os10g29230 | -0.80952 | - | -5.5413 | - |
| LOC_Os10g29260 | - | - | -6.65755 | -1.88759 |
| LOC_Os10g29320 | - | - | -7.26534 | - |
| LOC_Os10g29750 | 1.67158 | 1.196953 | -5.74828 | -1.1392 |
| LOC_Os10g29790 | - | - | -4.80601 | - |
| LOC_Os10g29810 | - | - | -7.27964 | - |
| LOC_Os10g29950 | -1.41566 | - | -5.57395 | - |
| LOC_Os11g02070 | 1.726513 | -1.01727 | -2.90559 | 1.032131 |
| LOC_Os11g24550 | 3.525898 | 0.320487 | -7.6051 | -3.75259 |
| LOC_Os11g40220 | - | - | -2.68086 | - |
| LOC_Os11g40490 | - | - | -6.63182 | - |
| LOC_Os11g40680 | -0.29804 | 0.827338 | -9.65893 | - |
| LOC_Os11g41290 | - | - | -6.52144 | - |
| LOC_Os11g41300 | 0.622043 | 1.633064 | -8.21435 | - |
| LOC_Os11g41350 | -0.70589 | 1.633064 | -10.7433 | - |
| LOC_Os11g45560 | - | 1.633064 | -5.32641 | -1.88759 |
| **E3 ubiquitin ligase RING** | | | | |
| LOC_Os01g03100 | -0.14787 | -0.06095 | -1.26191 | 0.14238 |
| LOC_Os01g38720 | - | -2.25736 | -5.56595 | - |
| LOC_Os01g50750 | 0.09729 | -1.5298 | -4.8966 | -2.02546 |
| LOC_Os01g73000 | -0.26822 | 0.204843 | 1.351771 | 0.046313 |
| LOC_Os02g36320 | 2.480657 | 1.085488 | -6.20006 | -4.70866 |
| LOC_Os02g46100 | -0.68528 | -0.03766 | -1.18592 | -0.50659 |
| LOC_Os02g52210 | 2.337276 | 2.268056 | 3.369565 | 0.700135 |
| LOC_Os02g54830 | -0.23029 | -0.59438 | -1.4369 | -0.44933 |
| LOC_Os03g05270 | -0.47065 | 0.507646 | -1.70687 | 0.944685 |
| LOC_Os03g20870 | -1.77747 | -0.59495 | 5.156947 | 0.047662 |
| LOC_Os03g22080 | - | - | -5.62534 | -5.16033 |
| LOC_Os03g42780 | 1.545497 | -1.10856 | -6.75595 | -8.17923 |
| LOC_Os03g42790 | -0.80952 | -2.65102 | -6.57861 | -0.87084 |
| LOC_Os03g44810 | 0.679702 | -0.58854 | -1.29503 | -0.31777 |
| LOC_Os03g46570 | -0.43834 | -1.01956 | -2.05073 | -1.69851 |
| LOC_Os04g49550 | -0.59889 | -0.10485 | -2.11151 | -2.38269 |
| LOC_Os04g49700 | -0.31191 | -0.33795 | -2.57725 | -4.30675 |
| LOC_Os05g47900 | 0.548921 | -0.07904 | -1.13305 | -2.3165 |
| LOC_Os06g03580 | 0.103575 | -0.04716 | 1.273517 | -0.12597 |
| LOC_Os06g06450 | -0.42063 | 0.435904 | -1.3362 | -0.73132 |
| LOC_Os06g09310 | 0.269765 | 0.685892 | 2.186771 | -1.26538 |
| LOC_Os06g11450 | -0.59102 | 0.636825 | 1.861668 | -0.83787 |
| LOC_Os06g51210 | - | 0.196097 | -5.28847 | 0.109747 |
| LOC_Os07g42610 | -0.20615 | -0.58303 | 1.748986 | -0.70783 |
| LOC_Os07g43840 | - | - | -5.36128 | - |
| LOC_Os07g43850 | - | - | -4.98062 | 0.826182 |
| LOC_Os07g47590 | -0.15421 | 0.549399 | 1.111605 | -2.15898 |
| LOC_Os08g03260 | 1.65153 | - | -5.97779 | -4.15307 |
| LOC_Os08g14320 | 0.12473 | 0.031322 | -1.55669 | -2.63424 |
| LOC_Os08g35060 | - | - | -5.84681 | -3.61012 |
| LOC_Os08g35070 | - | -1.2871 | -8.56593 | -3.96151 |
| LOC_Os08g37760 | -0.11473 | -0.36114 | -3.36497 | -0.89919 |
| LOC_Os08g38600 | 0.513199 | 0.083143 | -1.5572 | -2.03984 |
| LOC_Os08g43480 | -0.2843 | -0.2337 | -1.20984 | -0.45924 |
| LOC_Os09g21120 | 0.327277 | 1.532501 | -2.54304 | 1.472729 |
| LOC_Os09g25190 | 0.224897 | -0.25034 | -1.2724 | 0.662992 |
| LOC_Os09g25200 | 0.143996 | 2.622456 | 5.991433 | 0.901252 |
| LOC_Os09g25220 | - | -2.82914 | -5.13592 | -1.06035 |
| LOC_Os09g26470 | - | - | -9.65852 | -5.14239 |
| LOC_Os09g29310 | -0.95323 | 0.015996 | 1.337512 | -0.76207 |
| LOC_Os09g38630 | - | 0.196097 | -4.62647 | - |
| LOC_Os09g38640 | - | -1.63229 | -1.35085 | -0.89574 |
| LOC_Os09g39190 | 0.902638 | 2.412493 | -3.53218 | 0.54849 |
| LOC_Os10g32750 | -0.00304 | - | -5.74028 | - |
| LOC_Os10g40490 | -0.41166 | -1.21847 | 4.468632 | -0.10806 |
| LOC_Os12g24390 | - | 6.721146 | 7.433224 | 1.74499 |
| **E3 ubiquitin ligase SCF/SKP** | | | | |
| LOC_Os01g27160 | 0.442047 | -0.12515 | -3.2457 | 0.751065 |
| LOC_Os01g50840 | -0.64226 | 0.750172 | -1.00205 | 0.382041 |
| LOC_Os01g52970 | 0.39302 | 0.152371 | -4.02625 | 0.553714 |
| LOC_Os01g55430 | 0.497868 | -1.35998 | -2.75029 | -3.88263 |
| LOC_Os02g11790 | 0.478298 | 0.738457 | -3.6787 | 1.239181 |
| LOC_Os02g13180 | - | - | -6.26611 | -4.32213 |
| LOC_Os02g21260 | 0.174746 | -0.63999 | -2.39712 | 0.402058 |
| LOC_Os02g33240 | 0.169121 | -2.25736 | -7.16574 | 0.404039 |
| LOC_Os02g33310 | 0.611499 | 0.059021 | -1.14077 | -1.65465 |
| LOC_Os02g35530 | -0.16072 | -0.48797 | -1.8092 | 0.758577 |
| LOC_Os02g56800 | - | 5.212369 | 2.407554 | 1.818661 |
| LOC_Os03g02560 | -0.1 | 0.04474 | -1.25579 | -3.91018 |
| LOC_Os04g19750 | -2.31573 | -0.90471 | -2.37056 | -0.03745 |
| LOC_Os04g33820 | -0.61295 | 1.024057 | -1.39632 | -0.21328 |
| LOC_Os04g36000 | - | - | -7.41594 | -1.88759 |
| LOC_Os06g02360 | - | - | -2.7002 | -0.61424 |
| LOC_Os07g05150 | - | -2.25736 | -1.46957 | - |
| LOC_Os07g05160 | 1.545497 | - | -2.8328 | - |
| LOC_Os07g05180 | - | - | -2.32731 | 0.899688 |
| LOC_Os07g06900 | -0.32359 | - | -7.12824 | 0.86598 |
| LOC_Os07g22680 | 1.120126 | -0.17964 | -1.28642 | -2.87708 |
| LOC_Os07g43200 | -1.26864 | 0.353387 | -5.59484 | - |
| LOC_Os07g43250 | 0.369269 | -0.27535 | -1.11765 | -4.39743 |
| LOC_Os08g09410 | 1.132241 | 1.964507 | -4.42268 | -1.57019 |
| LOC_Os08g09640 | -0.65998 | 0.770464 | -5.97305 | -7.40387 |
| LOC_Os08g28800 | - | -1.2871 | -2.84142 | - |
| LOC_Os08g28820 | - | - | -2.75064 | - |
| LOC_Os08g34860 | - | 3.21539 | -3.32533 | 0.374526 |
| LOC_Os08g43530 | -1.26864 | 5.881155 | 2.018585 | - |
| LOC_Os09g10200 | -0.90008 | -1.39188 | -1.96185 | -1.90197 |
| LOC_Os09g10230 | 2.480657 | - | -2.03811 | - |
| LOC_Os09g10260 | - | 0.196097 | -1.87074 | - |
| LOC_Os09g10270 | - | -1.24465 | -2.08313 | - |
| LOC_Os09g10300 | 2.57862 | -1.41146 | -4.41646 | -8.17923 |
| LOC_Os10g03870 | 0.081198 | 0.848554 | -2.61509 | -0.13294 |
| LOC_Os10g30200 | -0.05487 | -0.3719 | -1.75562 | 0.302717 |
| LOC_Os12g33220 | 1.980757 | 0.761329 | -3.92263 | -8.10908 |
| E2 ubiquitin ligase | | | | |
| LOC_Os01g13280 | 0.081417 | -0.12272 | -2.40112 | -0.76205 |
| LOC_Os02g16040 | 0.393672 | 0.108557 | 1.064049 | 0.997296 |
| LOC_Os02g48910 | -1.26864 | - | -5.08074 | -1.68377 |
| LOC_Os02g48920 | - | - | -9.66367 | - |
| LOC_Os02g48950 | 0.250046 | -1.40832 | -2.44675 | 0.374526 |
| LOC_Os05g06120 | 1.67158 | -1.60639 | -5.26734 | 0.899688 |
| LOC_Os09g12310 | -0.06388 | 0.19 | -1.3775 | 0.76567 |
| LOC_Os09g15320 | 0.087926 | -0.14795 | -1.16395 | 0.926666 |
| **Ubiquitin** | | | | |
| LOC_Os01g45400 | - | - | -2.9738 | 1.032131 |
| LOC_Os01g45420 | - | - | -1.75756 | - |
| LOC_Os03g15370 | 0.12321 | 1.794812 | 2.498643 | -2.4627 |
| LOC_Os05g38310 | 0.340135 | 2.634326 | -6.09589 | - |
| LOC_Os07g30640 | - | - | -11.3915 | -9.47187 |
| LOC_Os08g08760 | - | - | -11.0334 | -5.28979 |
| LOC_Os09g27930 | 0.315757 | 8.379156 | 1.999495 | -3.8836 |
| LOC_Os09g31019 | 1.545497 | 8.324273 | -1.92336 | -4.72723 |
| LOC_Os09g31031 | 0.455712 | 0.961123 | 1.857467 | -1.16516 |
| LOC_Os10g34960 | 1.545497 | 0.229771 | -5.18888 | -1.88759 |
| **26S protease** | | | | |
| LOC_Os06g39870 | 0.035917 | -0.27637 | -1.05183 | -0.01584 |
| LOC_Os06g49020 | -1.11363 | -0.59762 | -1.83148 | -0.09301 |
| LOC_Os07g03760 | -0.39996 | -0.56007 | -2.6752 | 1.051933 |
| LOC_Os08g41730 | 0.407539 | -0.54127 | -3.0919 | -2.92091 |

**Table S9.** Primers used in this study.

| **ID** | **Sequence** |
| --- | --- |
| **Primers for mapping and BAC clone screening** | |
| xch43-F | GAGTTGAGAAGGCTAAAACGACT |
| xch43-R | TGTTTCCCCTTCTGTTTGCC |
| xch48-F | CCATGATCACCTTTTTAGCACAA |
| xch48-R | AAGGAAAACGTGTGGTTGCA |
| xch56-F | ACAGCAGAGTACAAGAGGCA |
| xch56-R | AGCATTTGGATGAGTACTTTGTGT |
| xch57-F | TCAGCCTAGCACACACTGAA |
| xch57-R | CTCAGTAAGCATAGCAGAGAGC |
| xch35-F | AGACGAAAGACTAACCCTAGACT |
| xch35-R | TCAGGCCTTCTCGAGTTCTG |
| xch16-F | GCGGTTAGGGTTTCGTC |
| xch16-R | GCGTATTCAGTTGGTGG |
| SM1-5-F | CTCCGATCCCCTCATCAAGA |
| SM1-5-R | ATATTAGGGGTGGTTGGATGAC |
| xch95-F | ATTCCCTAGTGGTTGCAGCT |
| xch95-R | AGGCTTCAGCTCCGACTTAG |
| xch99-F | GCGTCAAACTGTGTCCAAGT |
| xch99-R | TGTCACCATTATAGCCACACCT |
| xch127-F | CAGTACTCAAAGGGTTGCGG |
| xch127-R | TTCCTTCATCAACTCTCGGC |
| xch7-F | GAGATAGTGGTGGAGGTGGATGC |
| xch7-R | GCACTTGTACTCCCATTTCTCAACC |
| xch27-F | CCCTGGATCTCAACACCCAT |
| xch27-R | AACTACTGCCAATGCCATGC |
| SM1-F | GACTTTCTAGCATTGCCCATAT |
| SM1-R | GGCGAAGGGGAATTGGAATAAC |
| xch27-F | CCCTGGATCTCAACACCCAT |
| xch27-R | AACTACTGCCAATGCCATGC |
| xch213-F | CGATGAAAACGGATCGCACT |
| xch213-R | GCATCCACTAACCCACGTTC |
| xch211-F | GTGGGTTAGTGGCTGCAAAA |
| xch211-R | GGGGTCGGATTGCCACAC |
| xch207-F | GTACCTCCTTCACTGGCTGT |
| xch207-R | CAAAAACCCTTCAACCCCCA |
| xch204-F | AAACCACCTCCCAACCCAAA |
| xch204-R | TCCCTCGACTCATATACCCCT |
| **Primers for RT‒PCR and qRT‒PCR** | |
| LUC-qPCR-F | ATCCATCTTGCTCCAACACC |
| LUC-qPCR-R | GCGACGTAATCCACGATCTC |
| Ubi-qPCR-F | AACCAGCTGAGGCCCAAGA |
| Ubi-qPCR-R | ACGATTGATTTAACCAGTCCATGA |
| SDGMS-qPCR-F | CGCCTGAAGGAAGAACTTGG |
| SDGMS-qPCR-R | GCTGTAGGAGGTGCAGAGAA |
| **Primers for 1978-bp insertion detection** | |
| 1978-bp-F | ATCTTCTTCACTGCGTCCCA |
| 1978-bp-R | GTACCTCCTTCACTGGCTGT |
| **Primers for RACE** | |
| RACE-5-L | CATCTGGACTGCTCTTGGTCGGCTTC |
| RACE-5-S | GGCTTCAACTTCACCAGAAA |
| RACE-3-L | CTCCTTCACTGGCTGTTCTTTCTTTGG |
| RACE-3-S | CTCCGTCCAATAGCAAAGG |
| **Primers for CRISPR/Cas9 mutant** | |
| SDGMS-ATGF | CTCACCTGCATCACCAAAGA |
| SDGMS-ATGR | TTTGCTATTGGACGGAGCTT |
| **Primers for SDGMS protein expression** | |
| PMAL-SDGMS-F | GCCTGCAGGTCGACTCTAGATTACACGTCCTTGACATGCTTCTG |
| PMAL-SDGMS-R | GCCTGCAGGTCGACTCTAGACTCCAGGACTCCTACACAAACA |
| SDGMSM1F | GTTCCGATGGAAAGACCTCGCGTTTGAGGCCTTTCATGCTAAAG |
| SDGMSM1R | GCATGAAAGGCCTCAAACGCGAGGTCTTTCCATCGGAACAG |
| SDGMSM2F | GGTCGTGACCATCTCAGCGTCCATCCGCTTCCCGCTCTTG |
| SDGMSM2R | CAAGAGCGGGAAGCGGATGGACGCTGAGATGGTCACGACCGGCAG |
| SDGMSM3F | GACCATCTCAGAATCCATCGCGTTCCCGCTCTTGCAGCGATGGG |
| SDGMSM3R | CGCTGCAAGAGCGGGAACGCGATGGATTCTGAGATGGTCAC |
| SDGMSM4F | GTGAGTTCAACGAAGCGGGCAAGTACTCTCGTGCTTTG |
| SDGMSM4R | CAAAGCACGAGAGTACTTGCCCGCTTCGTTGAACTCACAGGAAAATTTC |
| **Primers for measuring relative fungal amount** | |
| 28S-F | TACGAGAGGAACCGCTCATTCAGATAATTA |
| 28S-R | TCAGCAGATCGTAACGATAAAGCTACTC |
| **Primers for preparing *in situ* hybridization probes** | |
| SDGMSinsituF | TAATACGACTCACTATAGGGAGACAAGAAGCTCATGGAGGACG |
| SDGMSinsituR | ATTTAGGTGACACTATAGAAGAGACTTGCCCCATTCGTTGAAC |

**REFERENCES**

1. Shi X, Zeng HY and Xue YD *et al.* A pair of new BAC and BIBAC vectors that facilitate BAC/BIBAC library construction and intact large genomic DNA insert exchange. *Plant Methods* 2011; **7**:33-33.

2. Livak KJ and Schmittgen T. Analysis of relative gene expression data using real-time quantitative PCR and the 2-DDCt method. *Methods* 2001; **25**:402–408.

3. Weng A. a novel adenine-releasing assay for ribosome-inactivating proteins a novel adenine-releasing assay for ribosome-inactivating proteins. *J Chromatogr B Analyt Technol Biomed Life Sci* 2018; **1072**:300-304.

4. Wang P and Tumer NE. Pokeweed antiviral protein cleaves double-stranded supercoiled DNA using the same active site required to depurinate rRNA. *Nucleic Acids Res* 1999; **27**:1900-1995.

5. Weng XY, Wang L and Wang J *et al.* Grain number, plant height, and heading date7 is a central regulator of growth, development, and stress response. *Plant Physiol* 2014; **164**:735-747.

6. De Block M and Debrouwer D. RNA-RNA in situ hybridization using digoxigenin-labeled probes: the use of high-molecular-weight polyvinyl alcohol in the alkaline phosphatase indoxyl-nitroblue tetrazolium reaction. *Anal Biochem* 1993; **215**:86-89.

7. Kim D, Langmead B and Salzberg SL. HISAT: a fast spliced aligner with low memory requirements. *Nat Methods* 2015; **12**:357-360.

8. Anders. S, Pyl. PT and Huber. W. HTSeq-a Python framework to work with high-throughput sequencing data. *Bioinformatics* 2015; **31**:166-169.

9. Love MI, Huber W and Anders S. Moderated estimation of fold change and dispersion for RNA-seq data with DESeq2. *Genome Biol* 2014; **15**:550.

10. Tian T, Liu Y and Yan HY *et al.* agriGO v2.0: a GO analysis toolkit for the agricultural community, 2017 update. *Nucleic Acids Res* 2017; **45**:W122-W129.

11. Thimm O, Bläsing O and Gibon Y *et al.* MAPMAN: a user-driven tool to display genomics data sets onto diagrams of metabolic pathways and other biological processes. *Plant J* 2004; **37**:914-939.

12. Xie KB, Chen JP and Wang Q *et al.* Direct phosphorylation and activation of a mitogen-activated protein kinase by a calcium-dependent protein kinase in rice. *Plant Cell* 2014; **26**:3077-3089.
